# Living well after breast cancer randomized controlled trial protocol: evaluating a telephone-delivered weight loss intervention versus usual care in women following treatment for breast cancer

**DOI:** 10.1186/s12885-016-2858-0

**Published:** 2016-10-28

**Authors:** Marina M. Reeves, Caroline O. Terranova, Jane M. Erickson, Jennifer R. Job, Denise S. K. Brookes, Nicole McCarthy, Ingrid J. Hickman, Sheleigh P. Lawler, Brianna S. Fjeldsoe, Genevieve N. Healy, Elisabeth A. H. Winkler, Monika Janda, J. Lennert Veerman, Robert S. Ware, Johannes B. Prins, Theo Vos, Wendy Demark-Wahnefried, Elizabeth G. Eakin

**Affiliations:** 1 School of Public Health, The University of Queensland, Brisbane, Australia; 2 School of Medicine, Children’s Nutrition Research Centre, The University of Queensland, Brisbane, Australia; 3 Icon Cancer Care, Wesley Medical Centre, Brisbane, Australia; 4 Department of Nutrition & Dietetics, Princess Alexandra Hospital, Brisbane, Australia; 5 Mater Research Institute, University of Queensland, Brisbane, Australia; 6 Baker IDI Heart and Diabetes Institute, Melbourne, Australia; 7 School of Physiotherapy, Curtin University, Perth, Australia; 8 Institute of Health and Biomedical Innovation, Queensland University of Technology, Brisbane, Australia; 9 Institute for Health Metrics and Evaluation, University of Washington, Seattle, USA; 10 Department of Nutrition Sciences, University of Alabama at Birmingham, Birmingham, USA

**Keywords:** Breast cancer survivors, Physical activity, Diet, Nutrition, Lifestyle intervention

## Abstract

**Background:**

Obesity, physical inactivity and poor diet quality have been associated with increased risk of breast cancer-specific and all-cause mortality as well as treatment-related side-effects in breast cancer survivors. Weight loss intervention trials in breast cancer survivors have shown that weight loss is safe and achievable; however, few studies have examined the benefits of such interventions on a broad range of outcomes and few have examined factors important to translation (e.g. feasible delivery method for scaling up, assessment of sustained changes, cost-effectiveness). The Living Well after Breast Cancer randomized controlled trial aims to evaluate a 12-month telephone-delivered weight loss intervention (versus usual care) on weight change and a range of secondary outcomes including cost-effectiveness.

**Methods/design:**

Women (18–75 years; body mass index 25–45 kg/m^2^) diagnosed with stage I-III breast cancer in the previous 2 years are recruited from public and private hospitals and through the state-based cancer registry (target *n* = 156). Following baseline assessment, participants are randomized 1:1 to either a 12-month telephone-delivered weight loss intervention (targeting diet and physical activity) or usual care. Data are collected at baseline, 6-months (mid-intervention), 12-months (end-of-intervention) and 18-months (maintenance). The primary outcome is change in weight at 12-months. Secondary outcomes are changes in body composition, bone mineral density, cardio-metabolic and cancer-related biomarkers, metabolic health and chronic disease risk, physical function, patient-reported outcomes (quality of life, fatigue, menopausal symptoms, body image, fear of cancer recurrence) and behaviors (dietary intake, physical activity, sitting time). Data collected at 18-months will be used to assess whether outcomes achieved at end-of-intervention are sustained six months after intervention completion. Cost-effectiveness will be assessed, as will mediators and moderators of intervention effects.

**Discussion:**

This trial will provide evidence needed to inform the wide-scale provision of weight loss, physical activity and dietary interventions as part of routine survivorship care for breast cancer survivors.

**Trial registration:**

Australian and New Zealand Clinical Trial Registry (ANZCTR) - ACTRN12612000997853 (Registered 18 September 2012).

**Electronic supplementary material:**

The online version of this article (doi:10.1186/s12885-016-2858-0) contains supplementary material, which is available to authorized users.

## Background

Breast cancer is the most common invasive cancer diagnosed among women in developed countries and the second most common cause of cancer death [1]. High incidence and high overall survival (between 80 and 90 % relative 5-year survival in most developed countries [1]) have resulted in a growing number of breast cancer survivors worldwide. Addressing survivorship issues for these women is important for improving quality of life and health outcomes, and for reducing burden on the health care system.

Excess body weight, physical inactivity, and poor diet quality are prevalent among breast cancer survivors, both prior to and following diagnosis and treatment, with over 60 % of survivors overweight or obese; over 60 % insufficiently active; and, over 80 % consuming inadequate amounts of fruit and vegetables [2–5]. These factors have been associated with poor outcomes (breast cancer-specific and all-cause mortality) [6–9] and an increased risk of treatment-related side-effects [10–15]. Obesity management has been identified as a priority area for cancer survivors, with breast cancer survivors being a sub-group where an obesity link with cancer progression appears particularly important [16].

A small but growing number of weight loss intervention trials in breast cancer survivors [17–20] have shown that modest weight loss is safe and achievable, and can improve some treatment-related side-effects as well as women’s quality of life in the short-term. However, a number of gaps in this evidence base remain, including: i) understanding the benefits of weight loss across a broader range of outcomes, i.e., assessment of hard endpoints (such as survival), intermediate biomarkers, co-morbidities, and patient-reported outcomes; ii) evaluating interventions that are: feasible to deliver and implement in routine practice, convenient and flexible to the patient, and that result in sustained behavior and weight change; iii) assessing economic outcomes; and, iv) identifying sub-groups of the population who benefit the most from particular interventions to inform a personalised approach to weight management [16, 21]. Comparisons of interventions against usual care are still warranted, particularly when examining patient-reported outcomes and treatment-related side-effects, as these may naturally improve over time following treatment completion. Comparison of cost-effectiveness against current practice (i.e., usual care) also is needed to inform translation into practice and allocation of scarce health care resources.

The Living Well after Breast Cancer trial aims to address a number of these gaps. This randomized controlled trial is evaluating a telephone-delivered weight loss intervention versus usual care in women following treatment for breast cancer. Specifically, the trial aims to:

- evaluate the effect of the intervention compared with usual care on percent change in weight at end-of-intervention (primary outcome); and changes in body composition, bone mineral density, cardio-metabolic and cancer-related biomarkers, metabolic health and chronic disease risk, physical function, patient-reported outcomes (quality of life, fatigue, menopausal symptoms, body image, fear of cancer recurrence) and behaviors (dietary intake, physical activity, sitting time) (secondary outcomes);
- assess whether changes in the primary and secondary outcomes are sustained six months after the end of the intervention;
- evaluate the cost-effectiveness of the weight loss intervention compared to usual care;
- identify subgroups who achieve the greatest benefit from the intervention (based on demographic, social and clinical characteristics, cancer-related characteristics and genomic profiles); and
- explore mediators and moderators of the intervention on primary and secondary outcomes to understand how the intervention worked.

## Methods

### Study design

Living Well after Breast Cancer is a two-arm parallel group randomized controlled trial evaluating a 12-month telephone-delivered weight loss intervention versus usual care in women diagnosed with breast cancer. An overview of the study design and the schedule for enrollment and study assessments is shown in Table 1.

**Table 1:**
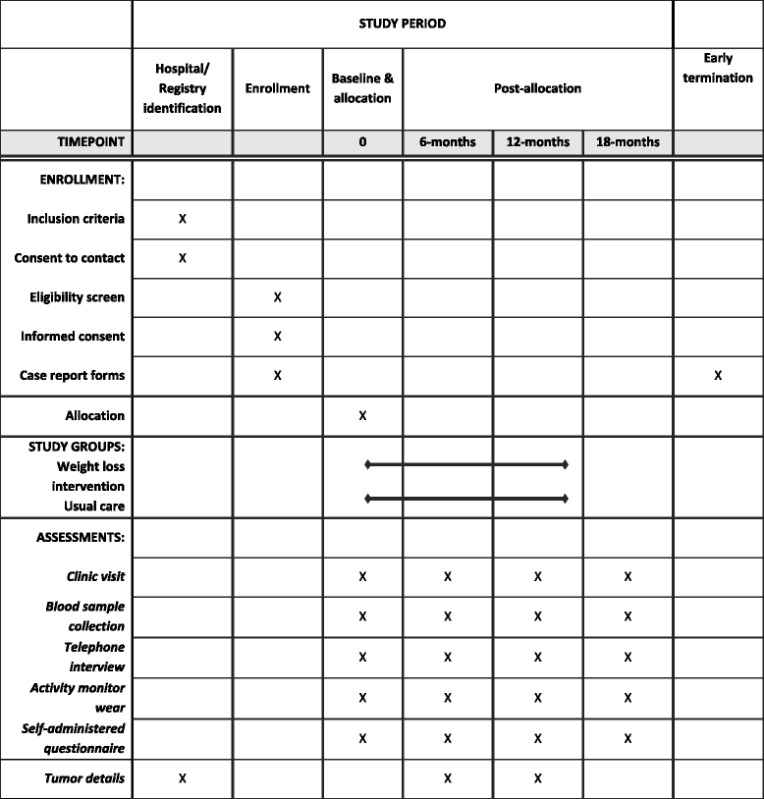
Schedule of enrollment, intervention, and assessment

Ethical approval was granted from the human research ethics committees of Greenslopes Private Hospital (12/26), Royal Brisbane and Women’s Hospital (HREC/12/QRBW/149), St. Vincent’s Health & Aged Care (13/02); and The University of Queensland Medical Research Ethics Committee (2012000944). A copy of the latest version of the study protocol approved by the ethics committees is included as Additional File 1. Approval was also granted from the Queensland Health Director General for accessing confidential information through the state-based cancer registry (RD004777). The trial was prospectively registered with the Australian New Zealand Clinical Trials Registry (www.anzctr.org.au; ACTRN12612000997853).

### Eligibility criteria

Inclusion criteria are: female, stage I-III breast cancer diagnosed within the previous 2 years (based on cancer registry pathology data), aged 18–75 years, body mass index (BMI) 25–45 kg/m^2^, and completed primary treatment (i.e., surgery, chemotherapy or radiotherapy). Continued hormonal treatment is permitted. Exclusion criteria are: pregnant; contraindications to participating in an unsupervised program (e.g., unstable heart disease, breathing problems requiring hospitalization in the last 6 months, undergoing dialysis, planning a knee or hip replacement in the next 6 months, regular use of a mobility aid); taking pharmacological doses of warfarin; greater than 5 % weight loss in previous 6 months; insufficient English to complete assessments and participate in the intervention; unable to travel to Brisbane to complete study assessments; or self-reporting depression, anxiety or other mental health condition as a current significant problem that would interfere with study participation. Women who develop a recurrence during the study period are withdrawn from the study.

### Participant recruitment

Participants were recruited through seven Brisbane hospital sites (Royal Brisbane and Women’s Hospital, Redcliffe Hospital, Mater Public Hospital, Mater Private Hospital, Greenslopes Private Hospital, North West Private Hospital, and Holy Spirit Northside Private Hospital; between October 2012 and June 2013), and through the state-based cancer registry (between July 2013 and December 2014). At hospital sites, nursing staff (e.g., breast care nurses, cancer care coordinators) provided potential participants with a study information packet during a routine consultation and briefly informed them of the study. The information packet contained a patient information brochure and consent to contact form with a reply-paid envelope. Interested women returned the form to their nurse or clinician or posted it directly to the research team. At one hospital site, nursing staff identified potentially eligible women from hospital records and posted the study information packet following confirmation of vital status against the state-based death register. Potentially eligible women diagnosed with breast cancer (based on age, stage of disease and residing within a 100 km radius of Brisbane) between 1 July 2013 and 30 June 2014, were identified through the Queensland Cancer Registry. Consistent with protocols for recruitment through the cancer registry, oncology care physicians identified from registry notifications, were first sent a letter to gain consent for patient contact. Physicians providing approval signed a letter informing their patient of the study, which was posted along with the patient information brochure, the consent to contact form and a reply-paid envelope. Additional recruitment methods included: posters placed in participating hospitals, word-of-mouth, and institution newsletters.

### Screening and consent

Women who are interested in hearing further about the study (i.e., return the consent to contact form) are posted an information sheet and consent form and are telephoned by study staff to explain the study, answer any questions, and screen for eligibility. Screening for BMI is based on self-reported height and weight. Women still undergoing primary treatment, but who are interested in the study, are contacted after treatment completion. Those who are eligible and interested provide signed informed consent.

### Randomization, allocation and blinding

The randomization sequence was generated using a computer-generated randomization program, with uneven block sizes ranging from four to 10 (www.randomization.com), by a staff member not otherwise involved with the study, and remained concealed from the study team. Following completion of baseline data collection, the project manager is notified of individuals’ allocation to study groups (randomized 1:1 into intervention or usual care) by the staff member responsible for the allocation sequence. In instances where participants have family or close friends already participating in the trial, participants are manually allocated (yoked) to the same study group as their family member or friend in order to prevent potential contamination. All assessors are blinded to participants’ study group allocation.

### Weight loss intervention

The weight loss intervention is based on clinical practice guidelines for overweight and obesity [22, 23] and recommendations for cancer survivors [24, 25], and has been previously pilot tested [26, 27]. The intervention uses a combined approach of increasing physical activity, reducing energy intake and behavior therapy (i.e., use of behavior change strategies), delivered by lifestyle coaches (accredited practising dietitians with additional study-specific training in exercise promotion), and aims for modest weight loss of between 5 and 10 %.

#### Intervention targets

##### Physical activity

Participants are encouraged to gradually increase activity, aiming for at least 210 min per week of planned (aerobic) activity at a moderate-to-vigorous intensity (30 min each day; if possible, increasing to 45–60 min/day), and 2–3 sessions of resistance exercise per week. These recommendations are consistent with physical activity guidelines for weight loss and weight loss maintenance, both generally [28] and specifically among cancer survivors [24, 25, 29]. Rather than being provided with a structured exercise program, participants identify planned activities that they enjoy and that can easily be incorporated into their lifestyle (e.g., walking, swimming, exercise classes), to meet the aerobic activity target. Participants who choose to do their resistance exercises at home are provided with detailed instructions and diagrams on home-based resistance exercises, including exercises using dumbbells and some without. In addition, participants are encouraged to increase their incidental/everyday activity (e.g., gardening, taking the stairs, housework), and reduce their sitting time (i.e., to get up and move at least every 30 min and to aim for no more than 2 h per day of screen time, outside of work hours). Participants are provided with a pedometer and encouraged to achieve 10,000 steps each day. This is consistent with evidence regarding the health consequences, notably cardio-metabolic, regarding high levels of sedentary time [30, 31], and the health benefits of increasing time spent in physical activities of any intensity [32, 33].

##### Dietary intake

Participants are encouraged to reduce energy intake by approximately 2000 kJ per day through a prescribed recommended kilojoule intake (between 5,000 and 7,500 kJ/day) based on age and baseline weight [34]. They are also encouraged to improve diet quality. Intervention strategies focus on portion control (by reducing portion size or number of serves) and reducing energy density, along with self-monitoring of food (and energy) intake. In addition, participants are encouraged to aim for: five serves per day of vegetables and two serves per day of fruit; total fat intake ≤30 % of energy; saturated fat intake <7 % of energy; and, limit alcohol intake to one standard drink per day (with at least two alcohol-free days per week), consistent with general dietary recommendations for weight management [22, 23] as well as specific recommendations for cancer survivors [24, 25].

##### Behavior therapy

Behavior change strategies and principles used to guide the intervention are evidence-based and derived from Social Cognitive Theory [35], which emphasizes self-monitoring, goal setting, problem solving, social support, stimulus control, positive self-talk and self-reward.

#### Intervention Protocol

The intervention is delivered entirely remotely, with no face-to-face contact, and involves: telephone coaching calls, a posted workbook and materials, and optional supportive text-messages. Intervention participants also receive a copy of a newsletter from the national breast cancer consumer organisation and a study newsletter after each assessment. In addition, participants are provided with written feedback following each assessment comparing their assessment results (body composition, dietary intake, physical activity, blood test results) to recommendations. Telephone coaches use baseline feedback to assess participants’ status at the commencement of the intervention and feedback from follow-up assessments to monitor their progress. The 12-month intervention includes: an initial intensive 6-month phase, followed by a 6-month extended care phase (see Table 2).

**Table 2:** Overview of intervention content

Intervention phase & call frequency	Purpose	Objectives
Initial phase		
6 weekly calls Optional text (SMS) messages	Building rapport Education Engagement Skill-building	• Program overview • Feedback on baseline assessment to build motivation • Understand importance of physical activity and diet in weight loss management • Build patient engagement through homework and self-monitoring • Use of behavior change skills and action plans: goal setting, self-monitoring, problem solving, rewarding success • Review progress, reinforce success & problem-solve barriers • Ongoing education
10 fortnightly calls Optional text (SMS) messages	Establish behavior change & achieve weight loss Skill-building	• Reflect on progress (changes made) and outcomes/benefits experienced • Progress goals • Continued use of behavior change skills: goal setting, self-monitoring, problem solving, rewarding success, managing slips • Ongoing education
Extended care phase		
6 monthly calls Optional structured text (SMS) messages	Consolidation Maintaining changes	• Encourage participants to direct sessions • Reinforce successes and review benefits • Behavior change skills: relapse prevention and maintaining motivation • Plans and strategies for maintaining weight loss and behavior changes

*SMS* short-messaging service

##### Initial intensive phase of intervention (months 1–6)

The focus of the initial intensive phase (weekly and fortnightly coaching calls over 6-months) is to build rapport, provide education about the importance of physical activity, healthy eating and weight management (by working through workbook content both during and in-between calls), encourage skill building through self-monitoring and goal setting, and work towards behavior change and weight loss (see Table 2). During this phase, participants receive up to 16 calls (6 × weekly calls, 10 × fortnightly calls) as well as optional text messages. Telephone call timing is scheduled based on participant preference (day vs. night). Participants also receive a detailed workbook, set of digital scales, measuring tape, pedometer, calorie counter book and self-monitoring diary, which is referred to throughout the intervention.

Lifestyle coaches use a motivational interviewing counseling style [36], with a semi-structured approach in relation to both call contact and content (e.g., the order in which intervention targets are addressed) based on participant preference. The protocol for each call includes: assessment of progress; problem solving; advice/education; collaborative goal setting; and development of a behaviorally-specific action plan. Call outlines and checklists are used by coaches to facilitate intervention fidelity. Fortnightly supervision meetings and audio-recording of randomly selected calls are used to monitor and maintain intervention fidelity, provide coaches with feedback throughout the intervention delivery period, and to discuss participants with particular challenges.

Participants also have the option to receive mobile phone text messages during this initial phase, starting from the second phone call. This aspect of the intervention is based on prior research on the use of mobile phone text messages for supporting and maintaining behavior changes and weight loss [27, 37]. In the initial phase, the participant, in consultation with the coach, determines the content and timing of the text messages. The texts are created (maximum of 160 characters) and sent using a web-based platform that enables the coaches to pre-schedule texts to send at specific times and days. Coaches received training on how to introduce the text messages and were provided with examples on the types of content they could include. All messages are personalized with the participant’s first name and signed off by the coach.

##### Extended care phase of intervention (months 7–12)

The focus of the 6-month extended care phase (6 × monthly calls) is to review progress, problem solve, and identify barriers and solutions to maintaining weight loss, physical activity and dietary changes. During this phase participants are encouraged to receive text messages between calls (regardless of whether they received texts during the initial phase). During the extended care phase, a more structured approach for text content and timing is used, with the texts designed to target specific behavioral skills: 1) prompting self-monitoring of weight; 2) behavioral goal setting; and, 3) prompting real-time behaviors (see Table 3 for examples). Each text type has a suggested frequency (Table 3), but is tailored to the participant’s preferences. The total number of text messages that can be received is in the range of two to 16 per fortnight (i.e. every two weeks). Participants who opt to receive the text messages during this phase first complete a scripted interview to collect information for tailoring the text messages. Text message content is tailored to: the names of the participant and coach, behavioral goals, rewards for reaching goals, identified barriers (and solutions) for reaching goals, preparatory behaviors to reach their goals, and outcome expectancies. Throughout this phase, tailoring information for the text messages is updated as requested by the participants or suggested by the coach.

**Table 3:** Examples of types of structured text messages that could be received during the extended care phase of the intervention (months 7 – 12)

Text message type	Behavior change strategies targeted	Example text messages	Frequency
Self-monitoring weight	Self-regulation; Satisfaction with perceived outcomes	Keeping track of ur weight is important 2 maintain progress & catch ‘slips’ Jane. Weigh yourself today & write it down in ur Living Well Diary. Jenny	1 per week or fortnight
Goal check	Self-regulation; Satisfaction with perceived outcomes	Hi Jane. Did u achieve ur goal 2 have 3 alcohol free days this week? Text me back yes or no so I know how u r going. Jenny	1 per week or fortnight per goal
Goal check reply	Self-regulation; Outcome expectancy; Satisfaction with perceived outcomes; Self efficacy; Social support	Wonderful news Jane! Remember how good u feel achieving ur goal & use this as motivation on ur ‘off’ days. Keep it up! Jenny	Only sent if participant responds to goal check
Behavior prompt		Think ahead Jane. U want 2 do 30 min on the treadmill 6× this week so make sure u set the alarm for the morning. Jenny	Up to 2 messages per week per goal

### Usual care

Participants in the usual care group continue to receive their standard medical care. In addition, these participants are posted materials after each of their study assessments (baseline, 6-months, 12-months, 18-months), which includes brief written feedback from their study assessment, a copy of a newsletter from the national breast cancer consumer organisation and a study newsletter. The feedback following study assessments is similar to that provided to intervention participants with the exception that participants’ results are not compared to national and study recommendations.

### Data collection

Data are collected from all participants at baseline, 6-months (mid-intervention), 12-months (end-of-intervention) and 18-months (follow-up after 6-months of no contact) by research staff blinded to participants’ study group (see Table 1). Each assessment involves: a clinic visit, blood sample collection, two telephone interviews, a self-administered questionnaire, and objective monitoring of physical activity by accelerometry for 7 days. The clinic visit includes objective measurements of height, weight, waist and hip circumferences, body composition, bone mineral density, blood pressure, and performance-based measures of physical function. Participants attend their local pathology collection centre after at least a 10-h fast to have blood samples (18 mL) taken by trained phlebotomists. Assays are conducted on fresh samples with serum samples also frozen at −80 °C for batch assays. Telephone interviews collect data on: physical activity levels, dietary intake, breast cancer-related information, and demographic and health characteristics. The self-administered questionnaire collects information on patient-reported outcomes and a range of constructs related to social-cognitive theory. Physical activity is also objectively measured via two activity monitors (one worn on the hip and one on the thigh), fitted at the clinic visit. Tumor characteristic data are obtained from pathology notifications within the cancer registry. Data related to intervention delivery are tracked in the study database. This includes data on call outcomes (call completion versus missed calls), call duration and call content (via a checklist of topics). A summary of study outcomes and data collection methods and tools is shown in Table 4. All staff received detailed training in data collection protocols and are blinded to participants’ study arm. Where appropriate, measurements are taken at least in duplicate.

**Table 4:** Primary and secondary outcomes and assessment methods

Outcome	Collection method	Assessment tool
Primary outcome		
Weight	Clinic visit	Tanita BWB-600 Wedderburn Scales
Secondary outcomes		
Anthropometry		
Waist circumference	Clinic visit	Non-expandable tape measure
Hip circumference	Clinic visit	Non-expandable tape measure
Body composition & densitometry		
Body composition (FM, FFM, LBM)	Clinic visit	Lunar Prodigy DXA - Total body and regional
Bone mineral density	Clinic visit	Lunar Prodigy DXA – Anterior-posterior lumbar spine (L1-L4); Bilateral proximal femur.
Cardio-metabolic & cancer-related biomarkers		
Glucose, lipids, HbA1c	Fasting blood test	Standard assays on fresh blood
Other cardio-metabolic and cancer-related blood markers	Fasting blood test	Stored serum, plasma, buffy coat
Metabolic health & chronic disease risk		
Blood pressure	Clinic visit	Welch Allyn 300 Series Vital Signs Monitor
Physical functioning		
Hand grip strength	Clinic visit	Smedley dynamometer
Timed chair stands	Clinic visit	5 stopwatch timed sit-to-stand transitions
Patient-reported outcomes		
Quality of life	SAQ	PROMIS Global Health Scale [50]
Fatigue	SAQ	Functional Assessment of Chronic Illness Therapy – Fatigue Scale (FACTIT-Fatigue) [51]
Menopausal symptoms	SAQ	Greene Climacteric Scale [53]
Body image	SAQ	Body Image and Relationships Scale (BIRS) [56]
Fear of cancer recurrence	SAQ	Concerns about Recurrence Questionnaire – 4-items (CARQ-4) [58]
Arthralgia	SAQ	Breast Cancer Prevention Trial Symptom Scale – Musculoskeletal Pain subscale [59]
Peripheral neuropathy	SAQ	Patient Neurotoxicity Questionnaire (PNQ) [61, 62]
Behavioral outcomes		
Dietary intake	Telephone interview	2 × 24-h dietary recalls
Physical activity	Objectively collected, Telephone interview	Actigraph GT3X+ tri-axial accelerometer Active Australia Survey [69]
Sitting time	Objectively collected	activPAL3^TM^ monitor

*DXA* Dual-energy X-ray Absorptiometry, *FFM* fat-free mass, *FM* fat mass, *HbA1c*, glycated hemoglobin, *LBM*, lean body mass, *SAQ*, self-administered questionnaire

### Primary outcome

The primary outcome is change in weight (% initial body weight). At each visit, weight is measured in duplicate to the nearest 0.1 kg, without shoes or heavy clothing, using calibrated scales (Tanita BWB-600 Wedderburn Scales, Australia), with the mean of the two values recorded.

### Secondary outcomes

Secondary outcomes include: anthropometry (waist and hip circumference), body composition, bone mineral density, cardio-metabolic and cancer-related blood markers, metabolic health (blood pressure and metabolic syndrome), performance-based measures of physical function, patient-reported outcomes (quality of life, fatigue, menopausal symptoms, body image, fear of cancer recurrence, arthralgia, chemotherapy-induced peripheral neuropathy), behavioral changes (dietary intake, physical activity, sitting time), and cost-effectiveness.

#### Anthropometry

Height is measured in duplicate to the nearest 0.1 cm using a stadiometer (Magnimeter, Raven Equipment, UK) at baseline. Waist and hip circumference are measured in duplicate to the nearest 0.5 cm using a non-expandable tape measure at the superior border of the iliac crest [38] and the greatest gluteal protuberance, respectively, following a normal expiration. A third measurement is taken if measures differ by more than 1.0 cm. The mean of all measurements taken is used.

#### Body composition & densitometry

Body composition and bone mineral density are acquired by Lunar Prodigy Dual-energy X-ray Absorptiometry (DXA; GE Medical Systems, LUNAR, Madison, WI, USA), using the manufacturer’s standard procedures. Daily calibration of the DXA is performed on the morning prior to each measurement using an aluminium spine phantom. All images are acquired and analysed by a trained technician (DSKB) using the manufacturer’s proprietary software (enCORE, version 14.1). Measures of body composition (fat mass (FM, g), percent fat (% region), fat free mass (FFM, g), and lean body mass (LBM, g; proxy for muscle mass)) are acquired from the total body scans. Total body scans provide measures of whole and regional body composition. Appendicular lean mass (LBM in arms and legs separated from trunk LBM) is calculated and used in the assessment of sarcopenia [39, 40].

Bone mineral density (BMD, g/cm^2^), bone mineral content (g) and bone area (cm^2^) for total body, anterior-posterior lumbar spine (LS 1–4) and bilateral proximal femur sites are measured. All BMD values are calculated as T- and Z-scores using the Geelong Osteoporosis Study reference database [41], for all sites.

#### Cardio-metabolic and cancer-related biomarkers

Glucose, glycated haemoglobin (HbA1c), total cholesterol, high density lipoprotein (HDL) cholesterol, low density lipoprotein (LDL) cholesterol and triglycerides are measured in fresh blood. HbA1c is measured from whole blood samples by the high performance liquid chromatography method (ion-exchange with ultraviolet detection; D-100 analyser, Biorad Laboratories, Hercules, CA, USA). Glucose, triglycerides, total cholesterol and HDL cholesterol are measured by an enzymatic colorimetric assay with Abbott c16000 Clinical Chemistry Analyzer (Abbott Diagnostics, Abbott Park, IL, USA). LDL cholesterol is calculated using the Friedewald equation [42].

Aliquots of serum, plasma and buffy coat are stored frozen at −80 °C to allow analysis of other relevant blood markers – for example insulin, adipokines (total and high molecular weight adiponectin, leptin), inflammatory markers (e.g., high-sensitivity C-reactive Protein), and genomic markers (e.g., microRNAs). All samples will be measured in duplicate with repeated samples from individuals assayed together to avoid batch variation.

#### Metabolic health & chronic disease risk

Blood pressure is measured seated using an automated blood pressure monitor (300 Series Vital Signs Monitor, Welch Allyn, Beaverton, OR, USA) with appropriately sized cuff. Measurements are taken in duplicate, with a third taken if the first two differ by ≥10 mmHg systolic or ≥6 mmHg diastolic, or if the first two readings are more than 140 mmHg/90 mmHg. The mean of the readings is recorded.

Comorbidities are assessed using the Charlson Comorbidity Index based on self-reported diagnosis of 13 conditions during the telephone interview [43, 44]. Participants also self-report if they have ever been diagnosed with hypertension [45]. Metabolic syndrome is classified according to the International Diabetes Federation worldwide consensus definition [46] and examined continuously [47, 48]. Current use of blood pressure, lipid lowering and/or diabetes medications are self-reported during the telephone interview. Comorbidities and medication use are assessed at baseline and each follow-up visit.

#### Performance-based measures of physical function

Hand grip strength and timed chair stands are used to assess upper and lower body function, respectively, as they have shown good to excellent reliability and high discrimination across different functional levels [49]. Bi-lateral hand grip strength (kg) is measured using a handheld dynamometer (Smedley, Scandidact, Denmark), with participants standing in neutral position with elbow flexed at 90°, the forearm in neutral and wrist held between 0 and 30° dorsiflexion and 0–15° ulnar deviation. Three measurements are conducted on each hand, alternating hands with a rest period in between measurements to prevent fatigue. Five chair stands are timed using a stopwatch. Participants are asked to perform, as quickly and ably as possible, five repetitions from a seated position to a fully standing position with arms crossed over their chest.

#### Patient-reported outcomes

##### Quality of life

is measured using the 10-item Patient Reported Outcome Measurement Information System (PROMIS) Global Health Scale which asks participants to evaluate their general health across five domains (physical function, fatigue, pain, emotional distress and social health) as well as general health perceptions. Items are scored into a Global Physical Health component and Global Mental Health component with higher scores indicating better functioning. The component scores have shown good internal consistency and good relative validity compared to EQ-5D [50].

##### Fatigue

is measured using the 13-item Functional Assessment of Chronic Illness Therapy – Fatigue Scale (FACIT-Fatigue) which assesses fatigue over the last seven days on a 5-point scale (0 = not at all to 4 = very much) [51]. Items are summed, giving a score of 0–52, with higher scores indicating lower fatigue. Fatigue is classed as present if the FACIT-Fatigue score is <34, corresponding to ICD-10 criteria for fatigue [52].

##### Menopausal symptoms

are measured using the 21-item Greene Climacteric Scale [53], which assesses the extent to which participants are affected by specified menopausal symptoms at present. Items are answered on a modified 5-point response scale from ‘none’ to ‘very severe.’ Responses of ‘severe’ and ‘very severe’ are collapsed to correspond to ‘extremely’ on the original 4-point response scale. Items are summed to create a total score as well as three subscale scores – psychological symptoms, somatic symptoms, vasomotor symptoms – with higher scores indicating more severe symptoms [53]. The total scale has shown excellent internal consistency, subscale scores have shown good two week test-retest reliability and are sensitive to change [54, 55].

##### Body image

is measured using the 32-item Body Image and Relationships Scale (BIRS), a scale developed specifically for women who have been diagnosed and treated for breast cancer [56]. The BIRS has shown good reliability, convergent and divergent validity, and sensitivity to change [56, 57]. Items are scored on a 5-point scale (1 = strongly disagree to 5 = strongly agree), with items summed to yield a total score and three subscale scores – strength and health; social barriers; sexuality and appearance – with higher scores indicating greater impairment.

##### Fear of cancer recurrence

is measured using the 11-point 4-item Concerns about Recurrence Questionnaire (CARQ-4) [58]. The four items are summed, giving a score of 0–40, with higher scores indicating greater fear. The CARQ-4 has shown good two week, test-retest reliability, and demonstrated concurrent and convergent validity with good correlations against the Fear of Cancer Recurrence Inventory and moderate correlations with measures of depression and anxiety [58].

##### Arthralgia

is measured using the Breast Cancer Prevention Trial Symptom Scale - Musculoskeletal Pain subscale, which includes three items assessing general aches and pains, joint pains and muscle stiffness over the past 4 weeks [59]. Items are scored on a 5-point severity scale from 0 ‘not at all’ to 4 ‘extremely,’ with scores from the three items averaged such that higher scores indicate a greater degree of being bothered by pain. The subscale has shown good internal consistency and is sensitive to change [59, 60].

##### Chemotherapy-induced peripheral neuropathy (CIPN)

is measured using the Patient Neurotoxicity Questionnaire (PNQ) [61, 62] in the subset of participants who were treated with chemotherapy. The PNQ includes two items assessing the presence and severity of sensory and motor disturbances over the past 7 days. Each item is rated on a 5-point scale from 0 ‘no neuropathy’ to 4 ‘severe neuropathy,’ with each item considered separately [63]. The PNQ scores show good concurrent validity against more detailed scales of CIPN [62] and are sensitive to changes over time [62, 63].

#### Behavioral outcomes

Physical activity and sedentary time are measured objectively at each assessment with the tri-axial Actigraph GT3X+ accelerometer (ActiGraph, Pensacola, Florida) and the activPAL3^TM^ monitor (PAL Technologies Limited, Glasgow, UK), each worn for seven consecutive days. Participants record sleep time and monitor removal times in a logbook. The Actigraph GT3X+ is worn positioned over the right hip via an adjustable elastic belt. Participants are asked to wear this monitor during all waking hours and to remove it only for sleep and during times the monitor could be damaged (e.g., during water-based activities). The activPAL is waterproofed, attached to the anterior mid-line of the right thigh using a hypoallergenic adhesive patch and worn continuously across the 7-day wear period, for 24 h per day (during waking and sleeping hours). Additional patches are provided to replace as necessary. The activPAL monitor records triaxial acceleration at 10Hz, from which thigh position, and the start and end of each period of time spent sitting/reclining, standing and stepping, are determined, along with transitions from sitting to standing and stepping speed. The activPAL has been shown to be a valid monitor for measuring sedentary behavior and is sensitive to detecting change [64]. The Actigraph GT3X+ monitor has shown acceptable relative validity when compared to oxygen consumption [65].

Raw GT3X+ data are collected at 30Hz and downloaded in Actilife (v 6.6.3). Both 10-s and 60-s epoch files are processed in SAS version 9.4. Non-wear time (estimated as blocks of ≥60 min of 0 counts per minute (cpm) with up to 2 min with counts 1–49 cpm) is removed [66]. Non-wear (invalid) days are also removed (<10 h wear or before/after the monitoring period based on the logbook). When quality controls (data visualisation and the logbook data) indicate participants wore the monitor to bed, self-reported sleep and naps are also removed. All minutes with ≥1952 cpm [67] are classed as moderate- to vigorous-intensity physical activity (MVPA), then summed for each day and averaged across valid days (i.e., ≥10 h of wear).

The activPAL data are downloaded using activPAL Professional 7.3.32 software (PAL Technologies Limited, Glasgow, UK). Recorded bouts of activity (sitting/reclining, standing and stepping) are processed in SAS version 9.4, using the monitor and logbook data with quality controls (data visualisation and cross-checking against the logbook). Unreported wake/sleep times are estimated by staff from times when movement first began/last ceased. All time during bouts that are ≥50 % during a self-reported sleep, removal or nap period are initially classed as sleep, removal, or a nap. Sleep periods are then adjusted to begin/end with the first/last sitting/reclining bout of ≥20 min duration during each period initially identified as sleep. Total sitting time (i.e., sitting/reclining during waking wear time) is summed for each day then averaged on valid days. Days are defined from wake on one day until wake the next day. Days are classed as valid if removals constituted <20 % of waking hours, and, when sleep/wake times are not reported, if waking wear time was ≥10 h. These methods are consistent with previous reports [68].

Physical activity is also self-reported using the Active Australia Survey, an 8-item questionnaire which assesses times spent walking, in moderate and in vigorous activities, and doing household and gardening activities, over the past week [69]. As per standard scoring protocols, self-reported MVPA is calculated as the sum of time spent walking, in moderate activities, and in vigorous activities (weighted by two), with truncation at 1680 min per week to reduce over-reporting. The Active Australia Survey has been shown to be valid, reliable, and responsive to intervention change [70–73]. The number of days and amount of time in the past week spent specifically walking for exercise [74], as well as undertaking strength or resistance based exercises, are also collected during the telephone interview.

Dietary intake is assessed using two unprompted 24-h dietary recall interviews (recalling one weekday and one weekend day) conducted using FoodWorks® Interview (version 1, 2009, Xyris Software, Brisbane, Australia), based on a 5-stage multi-pass method [75]. Participants are provided with a food model booklet to assist in estimating portion sizes. Energy and nutrient intakes are derived from dietary intake data using FoodWorks® Professional Edition (version 6, 2009, Xyris, Brisbane, Australia) nutritional analysis software, using the average of intakes from recalled days. Daily fruit and vegetable intake are also assessed using two items, which have been shown to be reliable and valid when compared to blood biomarkers [76, 77].

### Potential mediating and moderating variables

#### Demographics

Demographic and social characteristics collected during the baseline telephone interview include: highest educational attainment, employment status, household income, ethnicity, country of birth, marital status, children living at home and smoking status. Employment and smoking status are both re-assessed at each follow-up assessment. Age at baseline is determined from date of birth on pathology records. Participants’ residential postcode was used to assess area-level socio-economic position and geographical location/remoteness [78, 79].

At each assessment all participants self-report whether they have used any particular tools to assist with weight loss over the previous 6 months. This includes meal replacements, food delivery (pre-prepared meals) programs, other commercial weight loss programs (e.g. Weight Watchers), bariatric surgery or weight loss medications.

#### Cancer and treatment-related information

Cancer-related details are obtained from pathology records in the cancer registry and include: date of diagnosis; tumor size, type and histological grade; surgery details; receptor status (estrogen, progesterone and HER2/neu); and, lymph node involvement. Participants report menopausal status at diagnosis; treatments received (surgery, chemotherapy, radiation therapy, endocrine therapy, reconstructive surgery); treatment completion dates; and, presence (either current or past) of lymphedema.

#### Depression and anxiety

Participants self-report whether they have ‘ever been diagnosed with depression or anxiety.’ Depressive symptoms are also assessed using the 8-item PROMIS Short Form v1.0 – Depression 8b tool, which asks about negative mood, views of self and social cognition, decreased positive affect, and engagement over the last seven days on a 5-point scale (1 = never to 5 = always). Scores are summed (giving a score of 8–40) and converted to a T-score (M = 50, SD = 10) based on the U.S. general population [80]. This depression scale has demonstrated good reliability and validity [81].

#### Theory-based constructs

Data on constructs related to Social Cognitive Theory, and targeted as part of the intervention are collected using the self-administered questionnaire. Each of these constructs are assessed separately for physical activity and dietary intake, using items adapted from previous tools which have been described in detail elsewhere [37]. The constructs measured include outcome expectancies [82, 83], satisfaction with outcomes [84], self-regulation [85], self-efficacy [86, 87], social support [88], and perceived environmental opportunity [89–91].

### Adverse events

Data on adverse outcomes are collected at each follow-up assessment for all participants. Adverse outcomes are defined to participants as any new health problem such as: a breast cancer recurrence; other cancer diagnosis; diagnosis of other medical condition; period of hospitalisation; muscle injury or bone/joint problems; new symptoms; or, worsening of pre-existing conditions. Adverse events spontaneously reported either during intervention contacts or in between assessments are also recorded. The severity (5-categories ‘mild’ through to 'fatal') and relatedness to the intervention (5-categories ‘clearly not related’ through to ‘clearly related’) are recorded for all adverse events reported. The relatedness to the intervention is reported by the participant in discussion with the interviewer, as well as with consultation with the treating physician and principal investigator if needed. Unintentional weight loss is also assessed for all participants at each follow-up assessment.

### Retention strategies

A number of strategies are used to maximise retention over the 18-month study period. Of key importance is the development of good rapport with study staff (project manager, lifestyle coaches and assessors). Intervention calls and assessments are scheduled at times convenient to the participant. Participants travelling 50 km or further (round trip) to attend the clinic visit are provided with a gift card as reimbursement for their travel costs. Car parking costs are covered for all participants. Participants are contacted 2–4 weeks prior to their due date for the assessment to schedule their clinic visit and other aspects of their assessment. A 45-day window is allowed for the completion of the study assessment. Where necessary, participants who plan to be travelling, or are otherwise not available to complete all of the assessment during the scheduled window, coordinate with the project manager to complete their assessment earlier. All participants receive a study newsletter following each assessment and all participants are posted birthday cards. Feedback from the study assessments (e.g., bone mineral density DXA scan results) is provided to treating doctors as requested. Participants provide multiple contact details (e.g., mobile, home and/or work phone and email address) along with details of an emergency contact (family member or friend) to minimize loss to follow-up.

### Data management

Data sheets are stored in locked filing cabinets and electronic data are stored in password-protected files on a secure network system. Access to the study database is restricted. All data are double-key entered and checked for inconsistencies. Where appropriate, the database includes automatic range checks. In addition, all data are checked and cleaned prior to analysis.

### Statistical analyses

#### Sample size

The sample size calculation is based on the between-group difference in weight change (the primary outcome) 12 months after study entry, with a minimum clinically important difference (MCID) in weight change of 5 % of initial body weight [24]. Conservatively, assuming a standard deviation (SD) of change of 8.5 % [92], then with alpha = 0.05 and 90 % power we will need to collect 12-month data on 62 participants in each group (124 total). Allowing for 20 % attrition at 12 months, 78 per group (156 participants in total) will need to be recruited. With a sample size of 62 per group we will have 80 % power to detect effect sizes of 0.5 (i.e., minimum between group difference of half a SD) for the secondary outcomes. Of the secondary outcomes, only fatigue (FACIT-Fatigue) and hand grip strength (hand held dynamometer) have established MCIDs; 3 units and 3 kg, respectively [93, 94]. For these secondary outcomes, with our sample size of 62 per group we will have approximately 35 % power for detecting the MCID for fatigue and 96 % power for detecting the MCID for hand grip strength, assuming SDs of these outcomes in breast cancer survivors of 10.5 units [26, 95] and 4.5 kg [96], respectively.

#### Primary and secondary outcomes

Summary descriptive statistics for demographic, social and clinical data at baseline will be reported by allocated study treatment. Continuous data will be summarized descriptively using either mean and SD, or median and inter-quartile range, depending on the distribution of the variable of interest. Categorical data will be presented as frequencies and percentages. Comparisons between the treatment groups will be conducted to assess the degree to which comparability of randomization was achieved. The baseline between-group difference in the potentially influential confounding variables (stage of disease, menopausal status, treatment, pre-diagnosis weight, employment status and educational level) will be examined. If any of these variables are statistically significant at *p* < 0.001 they will be included as co-variables in all outcome analyses.

The primary study outcome is the change in weight (expressed as % of initial body weight) at 12 months. The mean difference between treatment groups will be calculated using linear mixed models with treatment group (intervention/usual care) and time (0/12/18 months) entered as fixed effects and patient entered as a random effect. We will include an interaction effect between treatment group and time in order to investigate change in weight. The corresponding 95 % Wald confidence interval and *p*-value will be reported. For secondary outcomes, the effect estimates will be presented as a mean difference, which will be calculated using a mixed effects linear regression model. For all models the corresponding 95 % Wald confidence interval and *p*-value will be reported. Statistical significance will be set at *p* <0.05 (two-tailed) and there will be no adjustment for multiple comparisons. All analyses will be intention-to-treat, with all evaluable data analysed according to the treatment group allocated.

To examine the sensitivity of results to attrition, analyses will be re-run after imputing missing data. The type of imputation will be decided after the characteristics of participants with missing data are compared against the characteristics of those with complete data. Either a single imputation method (using last observation carried forward) or a multiple imputation method (using chained equations) will be selected. Further, per-protocol analyses will be performed to compare those completing at least 75 % of intervention calls with those completing less intervention calls. Stratified analyses exploring effects on secondary outcomes based on amount of weight loss achieved (≥5 % weight loss vs. < 5 % weight loss) will be conducted.

#### Moderators and mediators of outcome

Exploratory analyses will be conducted to determine whether there is moderation or mediation of intervention effects. Moderator analysis will determine whether intervention effects differ across demographic (e.g., age, menopausal status) and breast cancer (e.g., receptor status/subtype, chemotherapy treatment, fear of cancer recurrence) characteristics and will be performed by considering the statistical significance of an interaction between a potential moderator and the intervention using a Wald test. Mediator analysis will determine whether theoretically-driven constructs and mechanisms for behavior change do in fact mediate the intervention effects. Potential mediators will be assessed using path analysis. Point estimates and bootstrap confidence intervals of path coefficients and the product of the mediated path coefficients will be used to determine the potency, certainty, and direction of any mediation effect.

### Cost-effectiveness

#### Intervention costs

Costs to deliver the intervention, not including the research/assessment components, are used in the cost analysis. They are tracked during trial implementation and include the cost of coach time, intervention materials (workbook, self-monitoring diary, pedometer, digital scale, measuring tape, kilojoule-calorie counter book) and related infrastructure (i.e., office space, telephones, computers and call costs).

#### Incremental cost-effectiveness/analysis

The cost-effectiveness analysis will present results as cost per health-adjusted life year (HALY) taking usual care as comparison. An existing micro-simulation model with a lifetime horizon (developed for the *ACE Prevention* project [97, 98]) will be adapted for this purpose. Epidemiological data will be updated to 2013 using estimates for Australia from the Global Burden of Disease study [99]. Demographic and health-related data collected at baseline and changes in weight and clinical biomarkers will be used to estimate lifetime risks of cardiovascular disease (based on sex, age, clinical biomarkers and body composition) using calibrated Framingham equations [100]. Other non-breast cancer health risks (diabetes mellitus, osteoarthritis, colon cancer, endometrial cancer and kidney cancer) will be modeled assuming that age-specific Australian average rates apply. Breast cancer recurrence risks and mortality will be modeled based on best available evidence at the time of conducting the modeling. We will take into account that after the 18-month assessment, weight is regained at a rate of 0.02–0.03 kg/m^2^ per month [101] and assume commensurate waning of the effects on clinical biomarkers. Years spent with disease will be valued less than healthy years by applying disability weights [102]. All of this will be used to calculate lifetime HALYs for all participants in the intervention and usual care groups. In both approaches, one-way sensitivity analysis and a combination of parametric and non-parametric bootstrapping will be applied. Net costs will be estimated from intervention costs and the modeled difference in health care costs in the remaining lifetime between the intervention and control groups. In both analyses, costing will take a health sector perspective that includes costs to patients and government. Costs associated with delivering the intervention (e.g. coach time, intervention materials) and costs incurred by participants (e.g., time) will be based on trial information. Costs associated with the development of the intervention will not be included. Costs of disease treatment will be taken from the Australian Institute of Health and Welfare’s Disease Costs and Impacts Studies. As per common convention, participants’ time will be valued at 25 % of the wage rate [103, 104].

## Discussion

The Living Well after Breast Cancer trial is the first large-scale randomized controlled trial of a weight loss intervention versus usual care in Australian breast cancer survivors. This trial adds to the international evidence in breast cancer survivors on the effectiveness of telephone-delivered weight loss interventions [17, 18, 105–107], which have greater potential for scaling-up into routine practice [21, 108]. Further, this trial will provide important evidence on the effect of weight loss on a broad range of secondary outcomes, some of which have not been examined in this population to date. Pertinent to the uptake of such interventions as part of routine follow-up care for breast cancer survivors, this trial will include an analysis on the cost-effectiveness of the intervention.

An additional strength of this study is the recruitment of a broad sample of breast cancer survivors through hospital clinics and the population-based cancer registry, including both pre- and post-menopausal breast cancer survivors and women with pre-existing comorbidities. Participants were recruited as close to the end of treatment as possible, which differs from many other weight loss intervention trials in breast cancer survivors, where participants were on average 3–5 years post-diagnosis at study baseline [18, 19].

The trial is powered based on the primary outcome of weight change. With the sample size it is powered to detect medium or larger effect sizes in secondary outcomes. For some secondary outcomes (e.g. fatigue), smaller effect sizes may be clinically meaningful but the trial will be underpowered to detect these. As a number of the secondary outcomes have not been previously examined in the context of a weight loss intervention in breast cancer survivors, results from this trial will provide preliminary evidence on the effect of weight loss on these outcomes. Due to the nature of the data collection procedures, recruitment was limited to women living within traveling distance of the capital city, therefore, results may not generalize to women living in regional and rural areas.

This trial will provide novel evidence on effectiveness and cost-effectiveness of a telephone-delivered weight loss intervention for breast cancer survivors. Findings from this trial will help address a number of gaps identified by the American Society of Clinical Oncology [16] and add to the evidence needed to inform the provision of weight loss interventions to breast cancer survivors as part of routine survivorship care.

## Additional file

Additional file 1: Study Protocol. (DOCX 159 kb)

## Abbreviations

ACTRN: Australian clinical trials registration number
BIRS: Body Image and Relationships Scale
BMD: Bone mineral density
BMI: Body mass index
CARQ-4: Concerns about Recurrence Questionnaire – 4 items
CIPN: Chemotherapy-induced peripheral neuropathy
Cpm: Counts per minute
DXA: Dual-energy X-ray Absorptiometry
FACIT: Functional Assessment of Chronic Illness Therapy
FFM: Fat free mass
FM: Fat mass
HALY: Health-adjusted life year
HbA1c: Glycated haemoglobin
HDL: High density lipoprotein
LBM: Lean body mass
LDL: Low density lipoprotein
LS: Lumbar spine
MCID: Minimum clinically important difference
MVPAm: Moderate-vigorous intensity physical activity
PNQ: Patient Neurotoxicity Questionnaire
PROMIS: Patient reported outcome measurement information system
SD: Standard deviation
