# Supplementary material for: Living well after breast cancer randomized controlled trial protocol: evaluating a telephone-delivered weight loss intervention versus usual care in women following treatment for breast cancer
Source: BMC Cancer. 2016 Oct 28;16:830. doi: 10.1186/s12885-016-2858-0 (PMC5086071; doi:10.1186/s12885-016-2858-0)
Supplement: Additional file 1: — Study Protocol. (DOCX 159 kb) [file 12885_2016_2858_MOESM1_ESM.docx]

Living Well after Breast Cancer

Randomised controlled trial of a telephone-delivered weight loss intervention for overweight and obese women following treatment for breast cancer

Registration number: ACTRN12612000997853

**Principal Investigator:**

| Name: | Dr Marina Reeves | | | | | |
| --- | --- | --- | --- | --- | --- | --- |
| Institution: | Cancer Prevention Research Centre, School of Population Health, The University of Queensland | | | | | |
| Address: | School of Population Health  The University of Queensland  Level 3, Public Health Building  Herston Rd  Herston QLD 4006 | | | | | |
| Contact details: | (ph) | 3346 4692 | (f) | 3365 5540 | (m) | 0418 982 201 |
| Email: | [m.reeves@sph.uq.edu.au](mailto:m.reeves@sph.uq.edu.au) | | | | | |

**Chief investigators**: Prof Elizabeth Eakin, Dr Ingrid Hickman, A/Prof Nicole McCarthy, Prof Theo Vos, Dr Eliza Whiteside

**Associate investigators**: Prof David Whiteman, Prof John Prins, Prof Wendy Demark-Wahnefried, Dr Sandi Hayes, Kathy Chapman, A/Prof Janette Vardy, A/Prof Monika Janda, Dr Lennert Veerman, Dr Elisabeth Winkler, Dr Sheleigh Lawler, Dr Genevieve Healy, Dr Brianna Fjeldsoe, Lorraine Woods, Fiona Evans

**Protocol Approval**

| **Protocol ID** | **Approval** | **Date** |
| --- | --- | --- |
| **PoCoG-2011-07** | PoCoG Scientific Advisory Committee Endorsement | **6/12/11** |
|  |  |  |
| **12/26** | Greenslopes Private Hospital Human Research Ethics Committee approval | **19/06/12** |
|  |  |  |
| **HREC/12/QRBW/149** | Royal Brisbane and Women’s Hospital Human Research Ethics Committee approval | **17/08/12** |
|  |  |  |
| **2012000944** | University of Queensland Medical Research Ethics Committee | **22/08/12** |
| **12/26** | Greenslopes Private Hospital Human Research Ethics Committee approval of amended protocol | **12/09/12** |
| **SSA/12/QNRC/37** | Metro North Hospital and Health Service Research Governance | **13/09/12** |
| **HREC/12/QRBW/149** | Royal Brisbane and Women’s Hospital Metro North Hospital and Health Service Research Governance | **14/11/12** |
| **RG-15AP** | Mater Health Services Research Governance | **27/11/12** |
| **ACTRN12612000997853** | Australia and New Zealand Clinical Trials Registry | **18/09/12** |
| **#13/02** | St Vincent’s Health & Aged Care Human Research Ethics Committee approval | **18/06/13** |
| **RD004777** | Queensland Health PHA approval to access identifiable data | **07/08/13;**  **16/08/13** |

#

# Table of Contents

[Study synopsis 5](#_Toc325965232)

[1. Study objectives 5](#_Toc325965233)

[1.1. Primary objective 5](#_Toc325965234)

[1.2. Secondary objective 5](#_Toc325965235)

[2. Background 6](#_Toc325965236)

[2.1. Justification/ Significance 6](#_Toc325965237)

[3. Study design 7](#_Toc325965238)

[3.1. Research question(s)/ aim(s) 7](#_Toc325965239)

[3.2. Hypotheses 7](#_Toc325965240)

[3.3. Study design 7](#_Toc325965241)

[4. Study population 8](#_Toc325965242)

[4.1. Inclusion criteria 8](#_Toc325965243)

[4.2. Exclusion criteria 8](#_Toc325965244)

[4.3. Withdrawal criteria 8](#_Toc325965245)

[5. Study procedures 9](#_Toc325965246)

[5.1. Participant recruitment 9](#_Toc325965247)

[5.2. Allocation method 12](#_Toc325965248)

[5.3. Study Groups 12](#_Toc325965249)

[5.3.1 Intervention group 13](#_Toc325965250)

[5.3.2 Usual Care group 13](#_Toc325965251)

[5.4. Blinding 13](#_Toc325965252)

[5.5. Duration of treatment/ study 14](#_Toc325965253)

[5.6. Assessment schedule and follow up 15](#_Toc325965254)

[5.6.1 Body Composition Assessment 15](#_Toc325965255)

[5.6.2 Blood Sample Collection 16](#_Toc325965256)

[5.6.3 Physical Activity Measurement 16](#_Toc325965257)

[5.6.4 Telephone Interviews 16](#_Toc325965258)

[5.6.5 Questionnaire 17](#_Toc325965259)

[5.6.6 Clinical and treatment information 18](#_Toc325965260)

[6. Safety reporting 18](#_Toc325965261)

[7. Outcomes/ Measures 18](#_Toc325965262)

[7.1. Primary outcome 18](#_Toc325965263)

[7.2. Secondary outcomes 18](#_Toc325965264)

[8. Statistical methods 19](#_Toc325965265)

[8.1. Sample size and justification 19](#_Toc325965266)

[8.1.1. Power for primary endpoints 19](#_Toc325965267)

[8.1.2. Power for secondary endpoints 19](#_Toc325965268)

[8.2. Early stopping 19](#_Toc325965269)

[8.3. Analytical plan 19](#_Toc325965270)

[8.3.1 Statistical analyses for primary outcomes 19](#_Toc325965271)

[8.3.2 Statistical analyses for secondary outcomes 19](#_Toc325965272)

[8.3.3 Sensitivity analyses 19](#_Toc325965273)

[8.3.4 Subgroup analyses 19](#_Toc325965274)

[8.3.5 Exploratory analyses 20](#_Toc325965275)

[8.4. Interim analyses 20](#_Toc325965276)

[8.5. Missing data 20](#_Toc325965277)

[9. Study administration and data management 20](#_Toc325965278)

[9.1. Protocol deviations 20](#_Toc325965279)

[9.2. Patient Identification 20](#_Toc325965280)

[9.3. Case Report Forms 20](#_Toc325965281)

[9.4. Data handling 20](#_Toc325965282)

[9.5. Record keeping 21](#_Toc325965283)

[9.5.1. Patient records 21](#_Toc325965284)

[9.5.2. Investigator/site records 21](#_Toc325965285)

[9.6. Training, monitoring and auditing 21](#_Toc325965286)

[9.6.1. Training 21](#_Toc325965287)

[9.6.2. Monitoring of recruitment sites 21](#_Toc325965288)

[9.6.3. Audits 22](#_Toc325965289)

[9.7. Cost assessment 22](#_Toc325965290)

[10. Regulatory considerations 22](#_Toc325965291)

[10.1. Participant consent 22](#_Toc325965292)

[10.2. Compliance with regulatory guidelines 22](#_Toc325965293)

[10.3. Ethical review 22](#_Toc325965294)

[11. References 24](#_Toc325965295)

[12. Appendices 26](#_Toc325965296)

# Study synopsis

| *Title:* | Living Well after Breast Cancer |
| --- | --- |
| *Sponsor:* | N/A |
| *Funding:* | NHMRC Project Grant APP1024739  $840,535 (2012-2015) |
| *Primary objective:* | To evaluate the telephone-delivered weight loss intervention compared to usual care, on its ability to promote and maintain weight loss, and to improve body composition, physical activity, dietary intake, clinical biomarkers of metabolic health (glucose, lipids, insulin, leptin, adiponectin, C Reactive Protein), genomic and DNA methylation-based biomarkers, blood pressure and patient-reported outcomes (quality of life, body image, fatigue, menopausal symptoms, fear of cancer recurrence). |
| *Rationale:* | Sixty percent of women with breast cancer are overweight and experience poorer outcomes (greater recurrence, reduced survival and increased risk of obesity-related chronic disease) compared to their healthy weight counterparts. Weight management support is recommended, but not integrated into clinical care. |
| *Study design:* | Randomised Controlled Trial |
| *Sample size (by treatment group):* | 80 per group (160 in total) |
| *Study arms (interventions and controls):* | Weight loss support; Usual Care |
| *Study endpoints (primary and secondary):* | Primary endpoint: 12 months (end of intervention)  Secondary endpoint: 18 months (end of study) |

# Study objectives

## Primary objective

To evaluate the telephone-delivered weight loss intervention compared to usual care, on its ability to achieve weight loss, and to improve body composition, physical activity, dietary intake, clinical biomarkers of metabolic health (glucose, lipids, insulin, leptin, adiponectin, C Reactive Protein), genomic and DNA methylation-based biomarkers, blood pressure and patient-reported outcomes (quality of life, body image, fatigue, menopausal symptoms, fear of cancer recurrence).

## Secondary objective

To assess whether changes in the primary and secondary outcomes are maintained 6-months after the end of the intervention.

To evaluate the cost effectiveness of the telephone-delivered weight loss intervention compared to usual care.

To explore mediators and moderators of the intervention effect on primary and secondary outcomes to understand how the intervention worked and for whom.

To explore whether certain genomic profiles are associated with greater weight loss success and weight loss maintenance.

# Background

## Justification/ Significance

Approximately 60% of Australian women diagnosed with breast cancer are overweight or obese [[1](#_ENREF_1)]. Excess body weight at diagnosis has been associated with an increased risk of distant recurrence and increased risk of death from breast cancer and overall mortality [[2](#_ENREF_2), [3](#_ENREF_3)]. This association has been observed in both pre- and post-menopausal women and is independent of tumour stage, adjuvant chemotherapy and endocrine treatment. Excess body weight also increases the risk of co-morbidities, such as type 2 diabetes and cardiovascular disease [[4](#_ENREF_4)]. International and Australian data show that the prevalence of co-morbidities is high among women with breast cancer (43% with at least one co-morbid condition)[[5](#_ENREF_5), [6](#_ENREF_6)] and that almost half of deaths are attributable to non-cancer causes [[7](#_ENREF_7), [8](#_ENREF_8)].

The mechanisms by which adiposity adversely affects breast cancer outcomes are unclear but likely due to a number of pathways [[9](#_ENREF_9)]. A number of metabolic biomarkers are known to be adversely affected by obesity – oestrogen, insulin, insulin-like growth factors, adipokines (e.g. leptin, adiponectin), inflammatory markers (e.g. tumour necrosis factor-alpha, interleukin-6, C-reactive protein [CRP]) [[9](#_ENREF_9)]. These biomarkers are all involved in a number of plausible biological pathways contributing to tumour-related angiogenesis and cellular proliferation [[9](#_ENREF_9)]. Recent epidemiological studies have linked a number of these metabolic biomarkers (i.e. insulin, leptin, adiponectin, CRP) to breast cancer recurrence, diseases-specific mortality and overall survival [[7](#_ENREF_7), [10-13](#_ENREF_10)]. Novel genomic markers may also play an important role in the association between excess body weight and poorer breast cancer outcomes [[14](#_ENREF_14)].

Only four randomised controlled trials have evaluated weight loss interventions for women with breast cancer [[15-18](#_ENREF_15)]. These trials evaluated shorter-term interventions with only one trial examining a 12 month intervention [[16](#_ENREF_16)]; none evaluated whether this weight loss was maintained following intervention completion; most recruit women a number of years after their diagnosis; and none have been conducted in Australia. Only two of these trials reported on changes in blood lipids (triglycerides, HDL-cholesterol), and one assessed changes in insulin and leptin (although these outcomes were underpowered) [[16](#_ENREF_16), [17](#_ENREF_17)]. Thus there is currently limited evidence on the effectiveness of weight loss interventions to achieve successful weight loss maintenance and to improve a range of metabolic and cancer-related biomarkers.

Two large-scale dietary-change (low fat and high fruit/vegetables, but not weight loss) intervention trials in women with breast cancer have followed women for 5-7 years to evaluate the impact on disease-free survival [[19](#_ENREF_19), [20](#_ENREF_20)]. One of these trials observed a beneficial impact on breast cancer recurrence (in concert with 3% weight loss) [[19](#_ENREF_19)]. Evidence from physical activity-only intervention trials demonstrates significant improvements in quality of life and treatment side-effects [[21-23](#_ENREF_21)]; along with some evidence for improvements in serum insulin and inflammatory marker concentrations [[24](#_ENREF_24)]. Evidence from observational studies suggests that post-diagnosis physical activity reduces risk of breast cancer recurrence, breast cancer mortality and overall mortality [[25](#_ENREF_25)]. The evidence suggests that improvements in physical activity and diet are likely to have independent beneficial impacts on a range of outcomes relevant to improving both the quantity and quality of survival in women with breast cancer.

The major Australian and North American cancer organisations now recommend that weight management should be a part of standard care for overweight women diagnosed with breast cancer [[26-29](#_ENREF_26)]. However, currently in Australia very little is done to provide weight management services as part of follow-up care; our own work shows that less than 25% of overweight and obese breast cancer survivors report receiving advice regarding weight management. Findings from this trial will inform the care of Australian women with breast cancer.

This study will evaluate the effectiveness of a weight loss intervention, compared to usual care, for women following treatment from breast cancer on a range of outcomes including weight and body composition, metabolic and genomic biomarkers and patient-reported outcomes. The intervention is delivered via the telephone to improve access to the intervention and allow for longer-term repeated contacts between the Coach and participant to improve the likelihood of successful weight loss maintenance. A cost-effectiveness analysis will inform decisions regarding the future delivery of the program.

# Study design

## Research question(s)/ aim(s)

The aims of the study are to:

1. To evaluate the telephone-delivered weight loss intervention compared to usual care, on its ability to promote and maintain weight loss, and to improve body composition, physical activity, dietary intake, clinical biomarkers of metabolic health (glucose, lipids, HbA1c, insulin, leptin, adiponectin, C Reactive Protein), genomic and DNA methylation-based biomarkers, blood pressure and patient-reported outcomes (quality of life, body image, fatigue, menopausal symptoms, fear of cancer recurrence).
2. To evaluate the cost effectiveness of the telephone-delivered weight loss intervention compared to usual care.
3. To explore mediators and moderators of the intervention effect on primary and secondary outcomes to understand how the intervention worked and for whom.
4. To explore whether certain genomic profiles are associated with greater weight loss success and weight loss maintenance.

## Hypotheses

1. Weight loss from baseline to 12-months follow-up, expressed as percent of initial body weight, will be at least 5% greater in the intervention group compared to the usual care group.

1b. Participants in the intervention group will report significantly greater improvements in all secondary outcomes (body composition, physical activity, diet, metabolic and genomic biomarkers, blood pressure, and patient reported outcomes) compared to the usual care group at the end of intervention (12-months).

1c. Differences observed between intervention and usual care groups for primary and secondary outcomes at 12-months will be maintained at 18-months follow-up.

2. The intervention will be cost-effective (less than $50,000 per disability-adjusted life year), compared to usual care.

Aims 3 and 4 are exploratory and not hypothesis testing.

## Study design

**Design:** This is a randomized controlled trial, with participants randomly allocated to one of two groups: 12-month weight loss support intervention or usual care.

**Data Collection:** Data collection points occur at baseline, 6-months (short-term), 12-months (end-of-intervention) and 18-months (maintenance post-intervention).

See Figure 1 for an overview of the study design

**Baseline 6 months 12 months 18 months**

Intervention Intensive phase: Maintenance Phase: Study newsletter Study newsletter

6 x weekly calls; 6 x monthly calls plus SMS (no telephone contact) (End of study)

10 x fortnightly calls

Usual Care Study newsletter & Study newsletter & Study newsletter & Study newsletter

brochures brochures brochures (End of study)

**Data**

**Collection X X X X**

**Figure 1:** Living Well after Breast Cancer study design

# Study population

The study population is women diagnosed with breast cancer who have recently completed treatment and are overweight or obese at the time of study enrolment. Potential participants will be identified through six Brisbane hospital sites (Royal Brisbane and Women’s Hospital, Redcliffe Hospital, Mater Public Hospital, Mater Private Hospital, Greenslopes Private Hospital, North West Private Hospital and Holy Spirit Northside Private Hospital) between October 2012 and February 2014. In addition, patients diagnosed with breast cancer from 1 July 2013 to 30 June 2014 will be identified through the Queensland Cancer Registry (via electronic pathology reports), or until the target sample size for recruitment has been achieved.

## Inclusion criteria

Eligibility will be based on the following inclusion criteria:

- Women
- Stage I-III breast cancer
- Aged 18-75 years
- BMI 25-45 kg/m^2^
- Finished primary treatment (i.e., surgery, chemotherapy and/or radiotherapy)

## Exclusion criteria

Women will be excluded if they meet any of the following criteria:

- Ductal carcinoma in situ (DCIS; stage 0) or distant metastases (stage IV)
- Diagnosed more than two years ago
- Pregnant
- Contraindications to participating in an unsupervised program (active heart disease, impaired mobility)
- Taking warfarin
- Greater than 5% weight loss in previous 6 months
- Insufficient English to complete assessments and participate in telephone-delivered program
- Unable to travel to Brisbane to complete study assessments
- Reporting depression/anxiety as a current significant problem that would interfere with study participation

## Withdrawal criteria

Participants will be withdrawn from the study if they report any of the following during the course of the study:

- Cancer recurrence or new cancer diagnosis
- Contraindications to participating in unsupervised physical activity, dietary or weight loss program (mobility issues, recent heart attack or angina, breathing difficulties requiring hospitalization or oxygen use, use of warfarin, pregnancy) – *Note: if a participant becomes pregnant during the course of the study, they may continue to receive intervention calls if they wish, with their doctor's approval*
- Moving interstate/overseas and therefore unable to complete study assessments

Participants are free to withdraw from the study at any point. If an intervention participant indicates that they wish to withdraw from the study they will be given the opportunity to withdraw from the intervention (and not receive any further telephone calls) but continue to complete the study assessments or to withdraw from the study completely and no complete any further intervention calls or study assessments.

Participants who choose to withdraw completely from the study will be asked to provide a reason for withdrawing which will be documented in the study database. Following this, participants will receive no further contact in relation to the study. Data collected from participants up until the point of withdrawal will be kept in the study database and used in data analysis. The study target sample size of 160 participants allows for attrition and therefore additional participants will not be recruited to replace withdrawing participants.

# Study procedures

## Participant recruitment

Recruitment will occur via nursing staff (Breast Care Nurses, Cancer Care Coordinators and other nursing staff) working at each of the participating hospitals. Staff will briefly inform each patient about the study (using a standard script; appendix a) and provide patients with a study information pack during a routine consultation (e.g. pre/post-surgery; beginning, mid or end of adjuvant treatment). In addition, interested cancer clinicians (surgeons, oncologists, radiologists) will provide patients at participating hospitals (Royal Brisbane and Women’s Hospital, Mater Adult Hospital, Mater Private Hospital, Redcliffe Hospital, Greenslopes Private Hospital, North West Private Hospital, Holy Spirit Northside Private Hospital) with a study information pack. The information pack will contain a Patient Information Brochure (appendix b) and Consent to Contact Form (appendix c) and reply-paid envelope. Women interested in finding out more information are instructed to complete the Consent to Contact Form and either hand it back to their nurse or clinician or post it back to the research team in the reply-paid envelope.

To identify women who were treated prior to recruitment commencing, nursing staff and volunteers under their direction at approving hospitals will send a letter to patients diagnosed within the previous two years about the study. Nursing staff and/or volunteers with approval to access hospital data will go through records of patients and make a list of patients who were diagnosed within the previous two years. A password-protected excel spread sheet containing the patients’ details (given names, surname, sex, date of birth, address, date of last contact) will be emailed from the hospital to the Registrar of the Health Statistics Unit for cross-checking against the Queensland Deaths Register – this will enable the identification of any deceased patients. Once the death matching has been completed, the password-protected spread sheet will be emailed back to the hospital staff identifying any deceased patients. These patients will then be excluded from the list of patients to be included in the study mail-out.

Hospital volunteers will then compile letters to be sent out to patients. This mail-out will include the following materials:

- Letter to the patient on hospital letterhead from their nurse
- Patient information brochure
- Consent to contact form
- Reply-paid envelope addressed to the research team

Study posters may also be placed in the appropriate clinic(s) at each of the hospitals. The posters instruct interested patients to contact the project manager directly. Other potentially eligible women may also hear about the study through word-of-mouth or media. These women may contact the project manager directly.

Interested woman who complete and return the Consent to Contact Form or contact the project manager directly will then be posted a Participant Information Sheet (appendix d) and Participant Consent Form (appendix e), which outlines in detail what the study is about, what participation would involve, benefits and risks of participation and provides contact details for the research team. A member of the research team will then telephone interested women within 7 days to screen for eligibility (appendix f), enable women to ask questions and determine their interest in participating. Women who would like to participate are encouraged to sign and return the Participant Consent Form (appendix e). Participation in the study will not commence until written informed consent is received.

Women who are still undergoing treatment (chemotherapy and/or radiotherapy) but who are potentially eligible and interested in the study will provide an estimated date of treatment completion which will be recorded in the study database. The women will then receive another telephone call after this date to again screen for eligibility, answer questions and determine interest (appendix f).

Women interested in taking part in the study are asked if they know anyone currently participating in the study during Screening and Recruitment call. If they have a close friend or relative already in the study who they maintain close contact with, women will be allocated to the same study group to prevent contamination.

Figure 2 provides an overview of the recruitment procedure.

Nursing staff (breast care nurses, cancer care coordinators) and interested cancer clinicians (surgeons, oncologists, radiologists) outline study & give patients from seven participating hospitals ‘Patient Information Brochure’ and ‘Consent to Contact Form’

Interested patients complete and return ‘Consent to Contact Form’ to project team or contact project manager directly

Patient details entered into study database, patient posted ‘Participant Information Sheet’ and ‘Participant Consent Form’

Project member telephones patient to screen for eligibility (finished treatment, breast cancer stage, contraindications to participation, BMI, sufficiently fluent in English) and gain informed consent to participate

Consent

Ineligible

Refusal

Treatment not completed

Signed ‘Participant Consent Form’ received

Patient estimated date for treatment completion recorded in database; recall after this date

Baseline assessment

Randomisation

Intervention

Usual care

**Figure 2.** Recruitment Flowchart

Identifying newly diagnosed breast cancer patients through the Queensland Cancer Registry

From 1 July 2013, a change in recruitment protocol will be implemented whereby recruitment will primarily occur through the Queensland Cancer Registry (QCR). Breast cancer patients diagnosed from 1 July 2013 to 30 June 2014, residing within a 100km radius of Brisbane, will be identified via electronic pathology reports held within the QCR. Recruitment will follow standard Queensland Cancer Registry protocol for recruitment of cancer patients following approval of a Public Health Act 2005 application by the Director-General of Queensland Health.

Patients diagnosed from 01 July 2013 to June 2014 and meeting the following criteria will be identified via QCR’s electronic pathology reports database:

- Breast Cancer (no evidence of DCIS or metastatic disease)
- Female
- Aged 25*-75 years (an existing study is identifying cancer patients aged up to 24 years through the Queensland Cancer Registry during the period of access requested)
- Residing within 100km radius of Brisbane CBD

Vital status will be checked for all identified patients by QCR staff using approved methods. Patients will then be sent a Patient Letter (on QCR letterhead), Patient Information Brochure and Consent to Contact Form with a reply-paid QCR envelope. Note: a different version of the Consent to Contact Form has been developed to be sent to patients identified through the QCR (Consent to Contact Form; version 2, modified 12 06 13). This version of the Consent to Contact Form instructs patients to complete the form to indicate either that they agree to be contacted about the study or that they do not wish to be contacted about the study. Patients who do not return the Consent to Contact form within 21 days will be sent a follow-up letter from the Registrar, on behalf of the researchers, as per standard QCR patient recruitment protocol. Patients who return the Consent to Contact form to state that they do not wish to be contacted will receive no further contact.

Once a patient’s Consent to Contact Form has been received and sighted by the Registrar, the Registrar will give permission for the researchers to take Consent to Contact Forms of patients who indicated that they agree to be contacted out of the QCR and back to the Cancer Prevention Research Centre (UQ). Interested women will be posted a Participant Information Sheet and Participant Consent Form and then telephoned by a member of the research team to screen for eligibility and elicit informed consent, as per the original recruitment protocol outlined above.

Amended hospital recruitment protocol

As patients diagnosed from 1 July 2013 to 30 June 2014 will be identified through the QCR, hospital recruitment protocol will be amended to allow for nursing staff and doctors within previously approved hospital sites to disseminate study information packs to breast cancer patients diagnosed prior to 1 July 2013. This will enable women diagnosed prior to 1 July 2013 returning to the hospital for adjuvant treatment or follow-up consultations to be informed about the study. In addition, study posters will continue to be displayed at approved hospital sites to allow interested women who have not been informed about the study by their nurse or doctor to contact the project manager to find out more information about the study.

## Allocation method

Participants will be allocated to either the Intervention group of the Usual Care group following completion of baseline data collection. A staff member who does not work on the study will create a computer-generated randomisation plan using uneven block sizes ([www.randomization.com](http://www.randomization.com)). This staff member will be notified when participants have completed baseline assessment and will allocate the participant IDs to either the usual care group or the intervention group based on the randomisation plan.

## Study Groups

Participants will be randomly allocated to one of two groups: weight loss support intervention group or usual care group.

### 5.3.1 Intervention group

The weight loss intervention will use a combined approach of increasing physical activity, reducing energy intake and behaviour change strategies. The intervention will be delivered entirely over the telephone by lifestyle coaches (who will be Accredited Practising Dietitians with additional study-specific training in exercise promotion). The intervention will be delivered over 12 months, with an initial intensive 6-month phase involving 16 calls (6 x weekly calls, 10 x fortnightly calls) followed by a 6-month maintenance-enhancement phase involving 6 monthly calls. Telephone calls will be scheduled based on participant preference (day vs. night). Participants will also receive a detailed workbook, set of digital scales, measuring tape, pedometer, kilojoule/calorie counter book and self-monitoring diary, which will be referred to over the 12-month intervention. Intervention participants will also be posted the same brochures and newsletter that the usual care participants receive (see below).

Participants will be encouraged to aim for weight loss of between 5-10% of their starting body weight. The physical activity component of the intervention includes both aerobic and resistance exercises, consistent with physical activity guidelines for cancer survivors. Women will be encouraged to slowly work towards a target of 30 minutes of moderate-level activity per day (210 minutes per week) and 2-3 sessions of resistance exercise per week. For diet, participants will be encouraged to reduce energy intake by 2000kJ. Participants will be prescribed a recommended kilojoule intake (between 5,000 – 7,500 kJ/day) based on age and baseline weight. Coaches will provide individualised advice including improving portion control, reducing energy density and eating regular meals along with kilojoule counting to achieve this energy reduction. In addition, participants will be encouraged to aim for total fat intake < 30% of energy, saturated fat intake < 7% of energy, 5 serves per day of vegetables and 2 serves per day of fruit. Both coach and participant will be provided with detailed feedback on the participant’s baseline and follow-up assessments (body composition, dietary intake, physical activity, blood test results) to monitor progress.

The behavioural component of the intervention will be guided by Social Cognitive Theory emphasising self-monitoring, goal setting, problem solving, social support, stimulus control, positive self-talk and self-reward. Coaches will use a motivational interviewing counselling style. The protocol for each call will include: assessment of progress; problem-solving; advice/education; collaborative goal-setting; and development of a behaviourally-specific action plan.

In addition, participants may choose to receive supplementary mobile telephone SMS (short-messaging service) from their Lifestyle Coach. As these SMS are optional, there is no set schedule of timing or content. These SMS are designed to provide additional support for achieving goals set during the telephone calls.

### 5.3.2 Usual Care group

Participants in the usual care group will continue to receive their standard medical care. In addition, these participants will be posted written materials after each of their study assessments (baseline, 6-months, 12-months, 18-months). These written materials will include brief feedback from their study assessment, off-the-shelf brochures and a study newsletter.

## Blinding

The group allocation sequence will be concealed from staff involved with participant recruitment (group allocation will be conducted by a staff member not involved with the study). Once allocated to the study group, the project manager and participants will not be blinded to the group allocation (as is the case with most lifestyle based interventions). Staff involved with data collection will remain blinded to the participants’ group allocation.

## Duration of treatment/ study

The Living Well after Breast Cancer study will continue recruiting patients into the study until a total of 160 participants have been randomised. After this recruitment target has been reached, no further enrolment will be possible and hospital sites will be informed to stop informing patients about the study. It is anticipated, that the accrual target will be reached by December 2014. The intervention and initial data collection is anticipated to be completed by December 2015. Furthermore, with a six-month follow-up, it is anticipated that final data collection will be completed by October 2016. See timeline below for an outline of anticipated milestones:

|  | **2012** | | | | **2013** | | | | **2014** | | | | **2015** | | | | **2016** | | | |
| --- | --- | --- | --- | --- | --- | --- | --- | --- | --- | --- | --- | --- | --- | --- | --- | --- | --- | --- | --- | --- |
| Develop study protocols, materials & database |  |  |  |  |  |  |  |  |  |  |  |  |  |  |  |  |  |  |  |  |
| Ethics & site specific approval |  |  |  |  |  |  |  |  |  |  |  |  |  |  |  |  |  |  |  |  |
| Participant recruitment |  |  |  |  |  |  |  |  |  |  |  |  |  |  |  |  |  |  |  |  |
| Data collection & intervention delivery |  |  |  |  |  |  |  |  |  |  |  |  |  |  |  |  |  |  |  |  |
| 6-month follow-up data collection |  |  |  |  |  |  |  |  |  |  |  |  |  |  |  |  |  |  |  |  |
| Analysis and reporting |  |  |  |  |  |  |  |  |  |  |  |  |  |  |  |  |  |  |  |  |

## Assessment schedule and follow up

Study assessments will be conducted at baseline, 6-months, 12-months and 18-months. Each study assessment will involve participants:

1. attending the body composition laboratory for an assessment;
2. attending a local pathology collection centre for a blood test;
3. wearing two activity monitors over seven (7) days (and returning monitors via post);
4. completing two telephone interviews;
5. completing a questionnaire.

| **Time point:** | **Hospital recruitment** | **Screening/ recruitment** | **Baseline assessment**  **(BL)** | **6-month assessment**  **(6M)** | **12-month assessment**  **(12M)** | **18-month assessment**  **(18M)** | **Early Termination** |
| --- | --- | --- | --- | --- | --- | --- | --- |
| **Inclusion criteria** | x |  |  |  |  |  |  |
| **Consent to contact** | x |  |  |  |  |  |  |
| **Informed consent** |  | x |  |  |  |  |  |
| **Exclusion Criteria** |  | x |  |  |  |  |  |
| **Case report forms** |  | x |  |  |  |  | x |
| **Randomisation/allocation** |  |  | x |  |  |  |  |
| **Body composition assessment** |  |  | x | x | x | x |  |
| **Blood sample collection** |  |  | x | x | x | x |  |
| **Activity monitor wear** |  |  | x | x | x | x |  |
| **Telephone interview** |  |  | x | x | x | x |  |
| **Questionnaire** |  |  | x | x | x | x |  |
| **Adverse events** |  |  |  | x | x | x |  |
| **Participant satisfaction (Intervention only)** |  |  |  |  | x |  |  |

At baseline, participants must complete the body composition assessment, have a blood sample taken, complete the physical activity measurement (wearing the hip and thigh monitors) and telephone interviews in order to be randomised into the study. Whilst it is desirable for participants to also complete the self-report questionnaire, and this will be encouraged, this data is not essential and participants can be randomised even if this is incomplete. Participants who fail to complete all the essential assessment components will not be randomised into the study.

### 5.6.1 Body Composition Assessment

The first component of each assessment to be completed is the Body Composition Assessment. The Body Composition Assessment is to be completed at the Body Composition Laboratory at the Royal Children’s Hospital. A laboratory technician, with the appropriate accreditation will conduct the assessment (appendix g).

The following data will be collected:

- Self-reported pre-diagnosis weight
- Height (stadiometer)
- Weight (digital scales)
- Waist circumference (non-expandable tape at top of iliac crest)
- Hip circumference (non-expandable tape at widest point of the buttocks)
- Blood pressure (Omron T8)
- Whole-body and segmental body composition - fat mass, fat-free mass (Lunar Prodigy DEXA scanner)
- Bone mineral density – hip and lumbar spine (Lunar Prodigy DEXA scanner)
- Physical functioning – hand grip strength (Smedley handheld dynamometer); timed chair stands (5 sit-to-stand repetitions)

At this assessment, participants will be provided with the pathology request form for the blood sample collection and two 5ml SST tubes to take with them to the Sullivan Nicholaides Pathology (SNP) Collection Centre, the activity monitor pack (including the hip and thigh monitors and express postage return pack), a food model booklet for use in the telephone interviews and the self-report questionnaire (with reply-paid envelope).

Body composition and bone mineral density will be measured by a DEXA machine. Having a DEXA scan done is similar to having an x-ray but has about 10% of the radiation of a chest x-ray.

### 5.6.2 Blood Sample Collection

Participants are given a Sullivan Nicolaides Pathology (SNP) request form to have 18ml of blood taken at their nearest Collection Centre within 2-3 days. Participants are instructed to fast for a minimum of 10 hours prior to having the sample taken.

Blood will be collected using standard aseptic procedures by trained phlebotomists. Samples will be spun at the SNP Collection Centre and then couriered to the SNP head office in Taringa, Brisbane. Some analysis will be performed on the fresh sample at SNP Pathology, including glucose, HbA1c, triglycerides and cholesterol profile. A 9ml sample will be couriered to the Princess Alexandra Hospital (PAH) Chemical Pathology where the sample will be centrifuged and the plasma, buffy coat and red blood cells aliquoted for freezing. The samples will be treated under usual strict temperature and blood handling procedures by trained chemical pathologists and kept in a secure area of the hospital. Members of the research team will collect the frozen samples from pathology on a weekly basis for longer-term storage in -80^o^c freezers on-site at the PAH. These freezers are kept in a secure area and accessible only to authorized staff.

Planned assays for metabolic biomarkers (insulin, leptin, adiponectin [total and high molecular weight], CRP [total and high sensitivity]) will be conducted by Mater Pathology and collaborators at Mater Medical Research Institute. Genomic and DNA methylation-based biomarkers will be assayed by collaborators at the Institute of Health and Biomedical Innovation at QUT.

### 5.6.3 Physical Activity Measurement

Participants are asked to wear a Thigh Monitor (activPAL3 inclinometer) and a Hip Monitor (Actigraph GT3X+ accelerometer) for 7 consecutive days. The activPAL3 is a small, lightweight and unobtrusive device worn on the front of the right thigh, about a third of the way between the top of the thigh and the knee, attached with an adhesive patch (Hypafix). The device is waterproofed by placing it in a nitrile finger cot and wrapping it in Tegaderm transparent film. The activPAL3 is worn continuously for the 7 days (i.e. during waking and sleeping hours). It records body posture and activity by monitoring the position of a person’s lower limb, and gives information on time spent sitting/reclining, standing and stepping as well transitions from sitting to standing. The GT3X+ accelerometer is a small, lightweight and unobtrusive device, worn on the right hip on an adjustable belt that records the time, duration and frequency of walking or running movements. The GT3X+ is worn during waking hours only and is removed for any water activities (showering, bathing, swimming etc.). During the 7 days the monitor is worn, participants complete a diary to record sleep time, the time the hip monitor is put on and removed for the day and within-day removal times for both the hip and thigh monitor.

### 5.6.4 Telephone Interviews

The telephone interview component consists of two unscheduled telephone interviews with participants (appendix h). The first telephone interview collects self-reported data on:

- physical activity levels - Active Australia Survey (eight items + two additional items on strength exercise)
- dietary intake – 24 hour dietary recall; fruit and vegetable intake; breakfast consumption (two items); perceived deprivation (two items)
- breast cancer related information – menopausal status at diagnosis; treatments received and date completed; reconstruction surgery; lymphoedema; Breast Cancer Network Australia (BCNA) membership
- demographic and health characteristics – age; employment status; educational level; income; marital status; ethnicity; smoking status; co-morbidities; use of blood pressure, lipid lowering and diabetes medications

The second telephone interview includes a second 24-hour dietary recall and the two perceived deprivation items. The two 24-hour dietary recalls should capture one week day and one weekend day.

The 24 hour dietary recalls are conducting using FoodWorks Interview Software, developed by Xyris Software, Brisbane, which uses the multi-pass method for recalling all foods and drinks consumed. Participants use the food model booklet provided to assist in estimating portion sizes.

During the final telephone interview for the study (at 18-months), participants will be asked if they give their consent to be recontacted in the future to see if they would be interested in continuing their participation in research in this area, should funding become available. They are only asked to provide consent to be contacted. If and when participants are contacted, they will be informed what would be involved in continuing participation and asked whether they choose to participate.

### 5.6.5 Questionnaire

The self-administered questionnaire collects information on patient reported outcomes (quality of life, fatigue, body image, menopausal symptoms, fear of cancer recurrence, arthralgia, peripheral neuropathy) and a range of constructs related to social-cognitive theory as well as sleep and depression, which will be examined to assess whether they mediate or moderate the relationship between the intervention and the primary and secondary outcomes (appendix i). The specific instruments included in the questionnaire are (version 3, amended 20/08/13):

- Outcome Expectancy for diet and physical activity – Diet items modified from Zunft et al., 1997; PA developed based on Rodgers & Brawley, 1991 & Williams et al., 2005
- Satisfaction with diet and physical activity outcomes – Developed for study based on outcome expectancy items
- Self-regulation for diet and physical activity – Modified from Petosa, 1993
- Self-efficacy for diet and physical activity – Diet modified Linde Barriers-based Self-Efficacy (for diet & PA) scale; PA modified Marcus et al., 1992
- Social support – Sallis Social Support for Eating & Exercise scales
- Perceived environmental opportunity for healthy eating and physical activity – Healthy eating modelled from Disdall et al., 2005 & Moore et al., 2008; Physical activity modified IPS-E, Inoue et al., 2008
- Sleep disturbance – Insomnia Severity Index (Q1-5)
- Quality of life – PROMIS Global Health Scale
- Fatigue – FACT-IT
- Depression – PROMIS SF-Depression 1.0
- Body image – PAL Relationships and Body Image Scale
- Menopausal symptoms – Greene Climacteric Scale
- Fear of cancer recurrence – Concerns about Recurrence Questionnaire (CARQ-4)
- Arthralgia (Breast Cancer Prevention Trial Musculoskeletal Pain Subscale)
- Peripheral neuropathy (Patient Neurotoxicity Questionnaire (PNQ))

The questionnaire is available for completion in either hard-copy (given to all participants attending for their Body Composition Assessment) or electronic format (LimeSurvey platform, hosted on the Faculty of Health Sciences secure server).

All intervention participants will also be given a short satisfaction questionnaire to complete at the end of the program (after 12-months) (appendix j). This questionnaire will ask about their overall satisfaction with the program, satisfaction with particular components, areas of participation they found difficult and suggestions for improvements to the program.

### 5.6.6 Clinical and treatment information

Clinical details including tumour size, type and histological grade, surgery details, lymph node involvement and hormonal status will be abstracted from pathology records, held at the Queensland Cancer Registry. A project officer will work on-site at the Queensland Cancer Registry to obtain this data from the Queensland Health electronic records database.

Participants will provide consent for treatment information (chemotherapy and/or radiotherapy cycle, agents etc.) to be collected from hospital medical records. This data will collected by a member of the research team following recruitment of all participants from each hospital. Collection of this data will be dependent on adequate resources for study staff to abstract this data from hospital medical records.

# Safety reporting

Adverse events (AEs) will be assessed for all participants at each follow-up assessment (6-, 12- and 18-month). In addition, all participants will specifically be asked about unintended weight loss (i.e. weight loss greater than expected based on changes made to physical activity and dietary intake). All AEs will be recorded on a standard Adverse Event Report Form (appendix k) and submitted to the chief investigator.

The chief investigator will determine the severity and relationship of the AE to the intervention and take appropriate action(s). Severity will be graded from 1 (mild) to 5 (fatal) based on the National Cancer Institute’s Common Terminology Criteria for Adverse Events (CTCAE) classification. The attribution of the AE to the intervention will be determined to be unrelated (unrelated or unlikely) or related (possible, probable or definite). AE Report Forms for participants reporting unintended weight loss will be reviewed by the CI in consultation with CI McCarthy (Medical Oncologist) to determine the appropriate course of action. The chief investigator will submit AE Report Forms to the Safety Monitoring Committee, which will oversee the monitoring of trial safety and AEs reporting for the study. The Committee will consist of three members including a cancer clinician and two behavioural scientists. The Chair of the committee will have appropriate experience or training in trial safety. All AEs will be reported to the chair of the committee, with serious AEs that may be related to the intervention reported within 48 hours of notification. The committee will meet via teleconference as needed.

# Outcomes/ Measures

## Primary outcome

- Weight loss (% initial body weight)

## Secondary outcomes

- Physical activity
- Dietary intake
- Waist and hip circumference
- Body composition and bone mineral density
- Blood pressure
- Clinical biomarkers of metabolic health: glucose, lipids, HbA1c, insulin, leptin, adiponectin, C Reactive Protein
- Genomic and DNA Methylation-based biomarkers
- Patient-reported outcomes: quality of life, fatigue, body image, menopausal symptoms, fear of cancer recurrence
- Cost-effectiveness

# Statistical methods

## Sample size and justification

### Power for primary endpoints

The sample size calculation is based on observing an average weight loss in the intervention group at the end of the intervention (12-months) of 7% of initial body weight[[30](#_ENREF_30)] and allowing for a small regain in weight in the intervention group from 12- to 18-months (+1.2%)[[31](#_ENREF_31)] and small changes in weight among the control group (approx ±1%).[[15-18](#_ENREF_15)] Thus, we expect that weight loss from baseline to 18-months follow-up, expressed as percent of initial body weight, will be at least 5% greater in the intervention group compared to the usual care group**.** Assuming a standard deviation of change of 8.5%[[16](#_ENREF_16)] and allowing 25% attrition at 18-months,[[32](#_ENREF_32)] 80 women per group (160 participants in total) will be needed to detect this minimum difference in weight loss between groups with 90% power and a type I error of 5% (two-tailed).

### Power for secondary endpoints

With a sample size of 80 per group we will have at least 80% power to detect clinically meaningful between-group differences for physical activity, energy intake, body composition, waist circumference, leptin, adiponectin, quality of life and body image, and between 50% to 80% power for the remaining secondary outcomes.[[16](#_ENREF_16), [30](#_ENREF_30), [33-39](#_ENREF_33)]

## Early stopping

There are no safety endpoints. No criteria for early study termination.

## Analytical plan

### 8.3.1 Statistical analyses for primary outcomes

Data analysis will be performed using SAS 9.2. Repeated measures regression model, using linear mixed model will be fitted, for the primary outcome (percent of initial body weight lost), including main effects of group and time and the interaction of group by time. The models will use baseline weight as a covariate to reduce the residual standard error and account for regression to the mean. Potential confounding variables such as stage of disease, menopausal status, treatment and pre-diagnosis weight will be examined to determine whether they are associated with the outcome and if so, will be included as covariates in the model. Statistical significance will be set at p < 0.05 (two-tailed).

### 8.3.2 Statistical analyses for secondary outcomes

Similar models will be performed for each secondary outcome, using the baseline value of the outcome as a covariate.

### 8.3.3 Sensitivity analyses

Sensitivity analyses will be conducted using different assumptions for handling missing data. Sensitivity analyses for secondary outcomes will include using different cut-points for device-measured physical activity; different cut-points for over- and under-estimating energy intake.

### 8.3.4 Subgroup analyses

Further analyses will be performed to compare the following groups:

- Those completing at least 75% of intervention calls
- Those with pre-existing type 2 diabetes or elevated cardio-metabolic biomarkers (e.g. LDL-cholesterol, blood pressure) at baseline

### 8.3.5 Exploratory analyses

Exploratory analyses will be conducted to determine whether there is moderation or mediation of intervention effects using previously established methods. Moderator analysis will determine whether intervention effects differ across demographic (e.g. age, menopausal status) and breast cancer (e.g. chemotherapy treatment, fear of cancer recurrence) characteristics and will be performed by considering the statistical significance of an interaction between a potential moderator and the intervention using a Wald test. Mediator analysis will determine whether theoretically-driven constructs and mechanisms for behaviour change do in fact mediate the intervention effects. Potential mediators will be assessed using path analysis. Point estimates and bootstrap confidence intervals of path coefficients and the product of the mediated path coefficients will be used to determine the potency, certainty and direction of any mediation effect [[40](#_ENREF_40)].

## Interim analyses

Analysis of 6-month and 12-month outcomes will be conducted once all 6-month follow-up and 12-month follow-up data has been collected, respectively.

## Missing data

Characteristics of those with missing data (drop outs) and those with complete data as well as group allocation and reason for drop out will be assessed to determine whether data are missing completely at random (MCAR), missing at random (MAR) or missing not at random (MNAR). Depending on the mechanism of missing data (MCAR, MAR, MNAR) an appropriate analytic choice will be made (e.g. completers analysis, multiple imputation or selection models) taking into consideration both bias and preservation of variance.

# Study administration and data management

## Protocol deviations

As a general rule, there should be no deviations from study protocol. Should there be a need to deviate from the protocol, this will be discussed with the investigator team and approval sought. The version of this document will be updated with each change/deviation, and each change noted. The revised protocol (including the updated version and protocol number) will then be sent to all reviewing HRECs as an amendment.

## Patient Identification

All patients that return the consent to contact form will be automatically assigned a 3-digit study-specific participant identification number (starting at 100) when their information is entered into the study database. This identification number is used to label all information collected from participants during the course of the study.

## Case Report Forms

Standard data collection forms (identified by participant study number) will be used to record all data collected from participants as part of the study health assessments.

## Data handling

Data collection will occur at the following time points: baseline, 6-months, 12-months, 18-months. All assessment data (body composition assessment, CATI, self-report questionnaire) will be hard-copy recorded using the standard assessment record. Participants’ blood test results will be compiled into a report by SNP and sent to the project manager. Hard-copy assessment data is then entered into the study database. Data will be key-entered twice for verification of correctness and accuracy. Hard copy data collection forms are stored in a locked filing cabinet in the project manager’s locked office. The study database will be stored on a secure network drive and accessible only to authorised project staff members. Results from metabolic and genomic biomarker assays will be provided in an electronic spread sheet.

In addition, data will be collected for participants allocated to the intervention group throughout the 12-month intervention period. The hard-copy data will be kept in a participant case folder by their assigned Lifestyle Coach and entered into the study database. Participant case folders will be handed to the project manager at the completion of the intervention period.

## Record keeping

###

### Patient records

All study records will be stored at the Cancer Prevention Research Centre, The University of Queensland. All data collected as part of study assessments will be kept in paper copy and then entered into the study database. Paper copy records will be stored in a locked filing cabinet in the project manager’s locked office. The study database, which stores all information (identifiable and de-identified) collected throughout the study, is saved in a secure folder on a secure network drive. Access to the study database will only be granted to relevant project staff. Electronic records will be saved in a password protected folder on secure network drive.

All study records will be kept in a secure document storage facility at the University of Queensland for 15 years after the completion of the study, in accordance with The Australian Code for the Responsible Conduct of Research.

### Investigator/site records

As hospitals are only required to disseminate information packs to patients, no records will be held at the hospital sites, all records will be held at the Cancer Prevention Research Centre, The University of Queensland. Some hospital sites may elect to collect signed patient consent to contact forms for the project manager to collect on a weekly basis.

## Training, monitoring and auditing

### Training

Hospital recruitment

As the recruitment protocols have been designed to minimise the time required by hospital recruitment staff (Breast Care Nurses, Cancer Care Coordinators or other nursing staff), no formal training will be required. They will be given a copy of the Hospital Recruitment Procedures document (appendix a), which outlines what the study involves and what their role is, a copy of the patient materials to be disseminated and a script to guide them in discussing the study with their patients.

Assessment and intervention

All project staff undertaking participant assessments will be given an assessment manual outlining assessment protocols, measurement and data entry requirements. Lifestyle Coaches will be given a counsellor manual which outlines the intervention protocols, structure and delivery. In addition, a database manual has been prepared for all staff to follow when entering assessment/intervention data. Telephone interviews (CATI) and intervention counselling calls will be periodically audio-recorded and reviewed by one of the study investigators to ensure adherence to assessment protocols and fidelity of intervention delivery. Regular team meetings will be held to discuss assessment and intervention progress and address any issues that may arise throughout the study.

### Monitoring of recruitment sites

The project manager will contact the primary Breast Cancer Nurse or Cancer Care Coordinator at each hospital once a week (via telephone) to check on recruitment progress, ensure that the study continues to be appropriately introduced and promoted by nurses to potential participants, and to address any issues that may have arisen. Each site will be sent a monthly email to notify them of the number of patients from their hospital that have been recruited into the study (participant names will remain anonymous).

Team meetings will be held fortnightly with the investigator team and project staff to report on recruitment progress, both at the hospitals (consent to contact) and on-site (consent to participate).

### Audits

All data collected (body composition assessment, telephone interviews, self-report questionnaire, blood test results) will be key-entered twice for verification of correctness and accuracy. In addition, telephone interviews and intervention calls will periodically be audio-recorded and reviewed by one of the investigators to check for adherence to assessment/intervention protocols.

## Cost assessment

This study has been fully costed with all elements (e.g., project staff salaries, equipment, recruitment, assessment and intervention calls and materials) covered by the NHRMC Project Grant funding and in-kind support from Cancer Prevention Research Centre.

# Regulatory considerations

## Participant consent

The nature of the study, what participation will involve, benefits and known risks of participation will be explained to the patients in a written document (Participant Information Sheet) (appendix d) and verbally (Screening and Recruitment call). The Participant Information Sheet will address the participant’s right to informed consent and the obligations of the researchers under privacy legislation in concordance with individual institutional ethics committees.

Written informed consent will be gained from each participant **prior** to commencing participation in the study (appendix e). Consent will be documented in the study database (accessible only to authorised persons) and the paper copy will be stored securely in a locked filing cabinet in the project manager’s locked office where it will be available for future reference.

## Compliance with regulatory guidelines

This study will be conducted in compliance with:

- the National Health and Medical Research Council National Statement on Ethical Conduct in Human Research (2007)
- The Australian Code for the Responsible Conduct of Research (2007)

## Ethical review

The study proposal received ethical approval from the Greenslopes Private Hospital HREC to cover recruitment at Greenslopes Private Hospital and North West Private Hospital on 19/06/2012. Royal Brisbane and Women’s Hospital HREC approval was received on 17/08/2012 to cover the Royal Brisbane and Women’s Hospital, Redcliffe Hospital, Mater Public Hospital and Mater Private Hospital. Ethical approval was granted from the University of Queensland Medical Research Ethics Committee on 22/08/2012. St Vincent’s Health & Aged Care HREC approval was received on 18/06/2013 to cover Holy Spirit Northside Private Hospital.

An amendment to the study protocol and recruitment documents was submitted to Greenslopes Private Hospital HREC based on RBWH HREC feedback (approved 12/08/2012).

Approval will be gained from the Director-General of Queensland Health to cover recruitment of breast cancer patients through the Queensland Cancer Registry (via electronic pathology reports).

Metro North Hospital and Health Service Research Governance approval was received on 13/09/2012 to cover the study recruitment at Redcliffe Hospital and on 14/11/2012 to cover the study recruitment at Royal Brisbane and Women’s Hospital. Mater Health Services Research Governance approval was granted on 27/11/2012 to cover study recruitment at Mater Adult Hospital and Mater Private Hospital.

During the course of the study, the chief investigator is required to submit to the HRECs the following:

- amendments to the protocol,
- serious and unexpected adverse events and the outcome,
- specific site updates as agreed to by the investigator and respective HREC, and
- any additional information (e.g., unexpected serious adverse events reported by other sites, administrative changes to the protocol).

At the end of the study, the Investigator is required to inform the HREC in writing that the study has ended and no further activities regarding this protocol will be conducted at the site.

# References

1. Hayes, S.C., et al., *Lymphedema after breast cancer: incidence, risk factors, and effect on upper body function.* Journal of Clinical Oncology, 2008. **26**(21): p. 3536-3542.

2. Ewertz, M., et al., *Effect of obesity on prognosis after early-stage breast cancer.* Journal of Clinical Oncology, 2011. **29**(1): p. 25-31.

3. Protani, M., M. Coory, and J.H. Martin, *Effect of obesity on survival of women with breast cancer: systematic review and meta-analysis.* Breast Cancer Research and Treatment, 2010. **123**(3): p. 627-35.

4. Eyre, H., R. Kahn, and R.M. Robertson, *Preventing cancer, cardiovascular disease, and diabetes.* Circulation, 2004: p. 3244-3255.

5. Patterson, R.E., et al., *Medical comorbidities predict mortality in women with a history of early stage breast cancer.* Breast Cancer Research and Treatment, 2010. **122**(3): p. 859-865.

6. Thompson, B., et al., *Patterns of surgical treatment for women diagnosed with early breast cancer in Queensland.* Annals of Surgical Oncology, 2008. **15**(2): p. 443-51.

7. Irwin, M.L., et al., *Fasting C-peptide levels and death resulting from all causes and breast cancer: the health, eating, activity, and lifestyle study.* Journal of Clinical Oncology, 2011. **29**(1): p. 47-53.

8. Yancik, R., et al., *Effect of age and comorbidity in postmenopausal breast cancer patients aged 55 years and older.* JAMA, 2001. **285**(7): p. 885-92.

9. Sinicrope, F. and A. Dannenberg, *Obesity and breast cancer prognosis: weight of the evidence.* Journal of Clinical Oncology, 2011. **29**(1): p. 4-7.

10. Duggan, C., et al., *Associations of insulin resistance and adiponectin with mortality in women with breast cancer.* Journal of Clinical Oncology, 2011. **29**(1): p. 32-9.

11. Goodwin, P.J., et al., *Fasting insulin and outcome in early-stage breast cancer: results of a prospective cohort study.* Journal of Clinical Oncology, 2002. **20**(1): p. 42-51.

12. Ishikawa, M., J. Kitayama, and H. Nagawa, *Enhanced expression of leptin and leptin receptor (OB-R) in human breast cancer.* Clinical Cancer Research, 2004. **10**(13): p. 4325-31.

13. Pierce, B.L., et al., *Elevated biomarkers of inflammation are associated with reduced survival among breast cancer patients.* Journal of Clinical Oncology, 2009. **27**(21): p. 3437-44.

14. Tao, M.H., et al., *Body mass and DNA promoter methylation in breast tumors in the Western New York Exposures and Breast Cancer Study.* American Journal of Clinical Nutrition, 2011. **94**(3): p. 831-8.

15. de Waard, F., et al., *A feasibility study on weight reduction in obese postmenopausal breast cancer patients.* Eur J Cancer Prev, 1993. **2**(3): p. 233-8.

16. Jen, K.L., et al., *Improvement of metabolism among obese breast cancer survivors in differing weight loss regimens.* Obesity Research, 2004. **12**(2): p. 306-12.

17. Mefferd, K., et al., *A cognitive behavioral therapy intervention to promote weight loss improves body composition and blood lipid profiles among overweight breast cancer survivors.* Breast Cancer Res Treat, 2007. **104**(2): p. 145-52.

18. Shaw, C., P. Mortimer, and P.A. Judd, *A randomized controlled trial of weight reduction as a treatment for breast cancer-related lymphedema.* Cancer, 2007. **110**(8): p. 1868-74.

19. Chlebowski, R.T., et al., *Dietary fat reduction and breast cancer outcome: interim efficacy results from the Women's Intervention Nutrition Study.* Journal of the National Cancer Institute, 2006. **98**(24): p. 1767-76.

20. Pierce, J.P., et al., *Influence of a diet very high in vegetables, fruit, and fiber and low in fat on prognosis following treatment for breast cancer: the Women's Healthy Eating and Living (WHEL) randomized trial.* JAMA, 2007. **298**(3): p. 289-298.

21. Brown, J.C., et al., *Efficacy of exercise interventions in modulating cancer-related fatigue among adult cancer survivors: a meta-analysis.* Cancer Epidemiology, Biomarkers and Prevention, 2011. **20**(1): p. 123-33.

22. Ferrer, R.A., et al., *Exercise interventions for cancer survivors: a meta-analysis of quality of life outcomes.* Annals of Behavioral Medicine, 2011. **41**(1): p. 32-47.

23. Schmitz, K.H., et al., *Controlled physical activity trials in cancer survivors: A systematic review and meta-analysis.* Cancer Epidemiology, Biomarkers & Prevention, 2005. **14**(7): p. 1588-1595.

24. Winzer, B.M., et al., *Physical activity and cancer prevention: a systematic review of clinical trials.* Cancer Causes and Control, 2011. **22**(6): p. 811-26.

25. Ibrahim, E.M. and A. Al-Homaidh, *Physical activity and survival after breast cancer diagnosis: meta-analysis of published studies.* Medical Oncology, 2010.

26. Ballard-Barbash, R., et al., *Physical activity, weight control, and breast cancer risk and survival: clinical trial rationale and design considerations.* Journal of the National Cancer Institute, 2009. **101**(9): p. 630-43.

27. Doyle, C., et al., *Nutrition and physical activity during and after cancer treatment: an American Cancer Society guide for informed choices.* CA: A Cancer Journal for Clinicians, 2006. **56**(6): p. 323-53.

28. National Breast and Ovarian Cancer Centre (NBOCC), *Recommendations for follow-up of women with early breast cancer (Supplement to Clinical Practice Guideline for the Management of Early Breast Cancer, 2nd edition 2001)*, 2010: Sydney.

29. World Cancer Research Fund/American Institute for Cancer Research, *Food, Nutrition, Physial Activity, and the Prevention of Cancer: a Global Perspective*, 2007, AICR: Washington DC.

30. Pi-Sunyer, X., et al., *Reduction in weight and cardiovascular disease risk factors in individuals with type 2 diabetes: one-year results of the look AHEAD trial.* Diabetes Care, 2007. **30**(6): p. 1374-83.

31. Perri, M.G., et al., *Extended-care programs for weight management in rural communities: the treatment of obesity in underserved rural settings (TOURS) randomized trial.* Arch Intern Med, 2008. **168**(21): p. 2347-54.

32. Wadden, T.A., M.L. Butryn, and K.J. Byrne, *Efficacy of lifestyle modification for long-term weight control.* Obesity Research, 2004. **12 Suppl**: p. 151S-62S.

33. Demark-Wahnefried, W., et al., *Changes in weight, body composition, and factors influencing energy balance among premenopausal breast cancer patients receiving adjuvant chemotherapy.* Journal of Clinical Oncology, 2001. **19**(9): p. 2381-9.

34. Digenio, A., et al., *Comparison of methods for delivering a lifestyle modification program for obese patients: a randomized trial.* Annals of Internal Medicine, 2009. **150**(4): p. 255-262.

35. Eakin, E.G., et al., *A randomized trial of a telephone-delivered exercise intervention for non-urban dwelling women newly diagnosed with breast cancer: Exercise for Health.* Annals of Behavioral Medicine, (under review).

36. Fjeldsoe, B.S., Y.D. Miller, and A.L. Marshall, *MobileMums: a randomized controlled trial of an SMS-based physical activity intervention.* Annals of Behavioral Medicine, 2010. **39**(2): p. 101-11.

37. Speck, R.M., et al., *Changes in the Body Image and Relationship Scale following a one-year strength training trial for breast cancer survivors with or at risk for lymphedema.* Breast Cancer Research and Treatment, 2010. **121**(2): p. 421-30.

38. Valsamakis, G., et al., *Modest weight loss and reduction in waist circumference after medical treatment are associated with favorable changes in serum adipocytokines.* Metabolism: Clinical and Experimental, 2004. **53**(4): p. 430-4.

39. Varady, K.A., et al., *Degree of weight loss required to improve adipokine concentrations and decrease fat cell size in severely obese women.* Metabolism: Clinical and Experimental, 2009. **58**(8): p. 1096-101.

40. Shrout, P.E. and N. Bolger, *Mediation in experimental and nonexperimental studies: New procedures and recommendations.* Psychological Methods, 2002. **7**(4): p. 422-445.

# List of Appendices

Appendix a) Hospital Recruitment Procedures

Appendix b) Patient Information Brochure

Appendix c) Consent to Contact Form

Appendix d) Participant Information Sheet

Appendix e) Participant Consent Form

Appendix f) Recruitment Screening Script

Appendix g) Body Composition Assessment Form

Appendix h) Telephone Interview Script

Appendix i) Self-report Questionnaire

Appendix j) Participant Satisfaction Survey

Appendix k) Adverse Event Report Form
